# BACE1-cleavage of Sez6 and Sez6L is elevated in Niemann-Pick type C disease mouse brains

**DOI:** 10.1371/journal.pone.0200344

**Published:** 2018-07-06

**Authors:** Mirsada Causevic, Kristina Dominko, Martina Malnar, Lea Vidatic, Stjepko Cermak, Martina Pigoni, Peer-Hendrik Kuhn, Alessio Colombo, Daniel Havas, Stefanie Flunkert, Jessica McDonald, Jenny M. Gunnersen, Birgit Hutter-Paier, Sabina Tahirovic, Manfred Windisch, Dimitri Krainc, Stefan F. Lichtenthaler, Silva Hecimovic

**Affiliations:** 1 Laboratory for Neurodegenerative Disease Research, Division of Molecular Medicine, Ruder Boskovic Institute, Zagreb, Croatia; 2 German Center for Neurodegenerative Diseases (DZNE), Munich, Germany; 3 Institut für Allgemeine Pathologie und pathologische Anatomie, Klinikum rechts der Isar der Technische Universität München and Institute for Advanced Study, Munich, Germany; 4 QPS Austria GmbH, Grambach, Austria; 5 The Ken and Ruth Davee Department of Neurology and Clinical Neurological Sciences, Feinberg School of Medicine, Northwestern University, Chicago, Illinois, United States of America; 6 Department of Anatomy and Neuroscience, The University of Melbourne, Parkville, Victoria, Australia; 7 The Florey Institute of Neuroscience and Mental Health, The University of Melbourne, Parkville, Victoria, Australia; 8 Munich Cluster for Systems Neurology (SyNergy), Munich, Germany; 9 Neuroproteomics, Klinikum rechts der Isar, and Institute for Advanced Study, Technische Universität München, Munich, Germany; Nathan S Kline Institute, UNITED STATES

## Abstract

It is intriguing that a rare, inherited lysosomal storage disorder Niemann-Pick type C (NPC) shares similarities with Alzheimer’s disease (AD). We have previously reported an enhanced processing of β-amyloid precursor protein (APP) by β-secretase (BACE1), a key enzyme in the pathogenesis of AD, in NPC1-null cells. In this work, we characterized regional and temporal expression and processing of the recently identified BACE1 substrates seizure protein 6 (Sez6) and seizure 6-like protein (Sez6L), and APP, in NPC1^-/-^ (NPC1) and NPC1^+/+^ (wt) mouse brains. We analysed 4-weeks old brains to detect the earliest changes associated with NPC, and 10-weeks of age to identify changes at terminal disease stage. Sez6 and Sez6L were selected due to their predominant cleavage by BACE1, and their potential role in synaptic function that may contribute to presentation of seizures and/or motor impairments in NPC patients. While an enhanced BACE1-cleavage of all three substrates was detected in NPC1 vs. wt-mouse brains at 4-weeks of age, at 10-weeks increased proteolysis by BACE1 was observed for Sez6L in the cortex, hippocampus and cerebellum of NPC1-mice. Interestingly, both APP and Sez6L were found to be expressed in Purkinje neurons and their immunostaining was lost upon Purkinje cell neurodegeneration in 10-weeks old NPC1 mice. Furthermore, in NPC1- vs. wt-mouse primary cortical neurons, both Sez6 and Sez6L showed increased punctuate staining within the endolysosomal pathway as well as increased Sez6L and BACE1-positive puncta. This indicates that a trafficking defect within the endolysosomal pathway may play a key role in enhanced BACE1-proteolysis in NPC disease. Overall, our findings suggest that enhanced proteolysis by BACE1 could be a part of NPC disease pathogenesis. Understanding the basic biology of BACE1 and the functional impact of cleavage of its substrates is important to better evaluate the therapeutic potential of BACE1 against AD and, possibly, NPC disease.

## Introduction

Alzheimer's disease (AD) is the most common form of dementia and the most common neurodegenerative disorder [1, 2]. So far, no disease modifying therapies against AD are available. Only symptomatic treatment options are approved. Currently, about 30 million patients are suffering from AD worldwide. Due to an ageing population, it is estimated that by the year 2050 numbers will increase to 106 million AD patients [1, 2]. These numbers raise the concern of AD becoming an epidemic problem. The β-secretase, known as β-site amyloid precursor protein cleaving enzyme 1 (BACE1), plays a central role in AD pathogenesis as it initiates the production of (toxic) amyloid-β peptides (Aβ) that accumulate in AD brains [3]. BACE1 has become a prime therapeutic target for lowering Aβ and thus, treating AD, and clinical development of BACE1 inhibitors is being intensely pursued [4].

It has been shown that AD shares several key features with the rare, inherited, still untreatable, neurodegenerative disorder Niemann-Pick type C (NPC) [5, 6]. Common features include accumulation of intracellular Aβ and APP C-terminal fragments (APP-CTFs), accumulation of hyperphosphorylated tau, neurodegeneration, activation of astrocytes and microglia, and altered cholesterol metabolism. In contrast to AD, which is a complex disorder involving both genetic and environmental components, NPC is a monogenic autosomal recessive disorder caused by mutations in either *NPC1* or *NPC2* gene [7, 8, 9]. Both code for proteins involved in cholesterol transport. NPC can represent an innovative model in which a defect of a single gene could be used to dissect certain molecular aspects of complex AD. Due to similarities between NPC and AD, NPC disease has also been termed “juvenile Alzheimer’s disease”. However, these two neurodegenerative disorders also show clear distinctions. In contrast to AD, where neurodegeneration primarily occurs in hippocampus and cortex, while cerebellum seems to be protected, in NPC disease Purkinje cells in the cerebellum are the most affected cell types in the brain, while the hippocampus seems to be spared. Different vulnerabilities of brain regions (hippocampus vs. cerebellum) between these two neurodegenerative disorders may give us important clues about protective and/or vulnerable mechanism(s) of neurodegeneration and may also identify critical components in this process. Therefore, further studies of similarities and differences between AD and NPC are encouraged to increase our understanding of the molecular events taking place during the pathogenesis of these conditions, as well as our chances of finding disease-modifying therapies. In AD, for example, the pharmacological inhibition of β-secretase is one of the most promising therapeutic approaches which has entered clinical trial phase II/III [4]. However, recent discovery of multiple BACE1 substrates has raised the concern on potential side-effects that may occur upon BACE1-inhibition [10]. Indeed, prolonged inhibition of BACE1 has shown to impair synaptic plasticity [11, 12]. Thus, further studies are needed to elucidate the function and the effect of cleavage of BACE1 substrates to better evaluate therapeutic potential of BACE1 and foresee the side-effects of BACE1-inhibition.

We have previously reported that increased levels of Aβ and soluble APPβ (sAPPβ) fragments in NPC1-null cells are due to enhanced BACE1 processing of APP [13] which most likely involves cholesterol-mediated sequestration of both APP and BACE1 within the endocytic compartments [14] and/or within lipid rafts [15]. In line with the recent identification of additional BACE1 substrates [10, 16, 17], in this study, we tested whether enhanced BACE1-dependent proteolysis occurs in NPC1^-/-^ (NPC1) mouse brains and whether it is specific for APP or could also be observed for other BACE1 substrates that could be of relevance for disease pathology. Namely, we analysed processing and localization of seizure protein 6 (Sez6) and seizure 6-like protein (Sez6L) in NPC1 vs. wt mouse brains (cortex, hippocampus and cerebellum) at 4-weeks of age, to detect the earliest BACE1-mediated changes associated with the disease, and at 10-weeks of age to identify changes at the end-stage of disease. Sez6 and Sez6L were selected as being top BACE1 substrates [18] and nearly exclusively cleaved by BACE1 in neurons [10, 19] and not—like APP—additionally by an α-secretase [18, 20]. Although the function of Sez6/Sez6L proteins is unknown, both proteins may be involved in synaptic (dys)function [21, 22]–characteristic features of both AD and NPC diseases. In addition, these proteins were analysed due to the fact that patients with NPC disease exhibit seizures together with the loss of motor control, dementia and other neuropathological symptoms [9, 23]. We hypothesized that, upon NPC1-dysfunction, cholesterol-mediated sequestration of BACE1 within the endolysosomal system (where BACE1 is most active) causes enhanced cleavage of BACE1-substrates, and that increased BACE1-mediated proteolysis may also contribute to the pathological features of NPC disease.

## Materials and methods

### NPC1 mouse model

A mouse model of NPC disease, the BALB/cNctr-*Npc1*^*m1N*^/J (stock number 003092), were purchased from the Jackson Laboratory, Bar Harbor, Maine, USA, and were housed at the JSW Lifesciences GmbH / QPS Austria GmbH animal facility in accordance with the EU Directive 2010/63/EU for animal experiments. Mice were maintained on a 12 hour light/dark cycle with access to water and a standard mouse diet *ad libitum*. Female and male NPC1^+/-^ mice were mated to generate NPC1^-/-^ (NPC1) and NPC1^+/+^ (wt, control) mice as NPC1^-/-^ (NPC1) mice are not fertile. Mice were genotyped according to already published protocols (http://jaxmice.jax.org/strain/003092.html). Mouse genotype was further confirmed by using sodium dodecyl sulphate-polyacrylamide gel electrophoresis (SDS-PAGE)/Western blotting, and immunoblotting with an anti-NPC1 antibody (ab134113; Abcam). All the experiments on these mice were approved by the Ministry of Agriculture of the Republic of Croatia and by the Bioethics Committee of the Ruder Boskovic Institute.

All mice were deeply anesthetized by isoflurane inhalation anesthesia (Isoba, Essex). Afterwards, mice were transcardially perfused with 0.9% saline solution. Brains were removed and hemisected; left hemispheres were immediately cut into cerebellum, hippocampus, cortex and remaining brain and snap-frozen in liquid nitrogen and stored at -80°C, whereas right hemispheres were immersion fixed in fresh 4% paraformaldehyde in phosphate-buffered saline (PBS) for one hour and then transferred to a 15% sucrose solution for 24 hours to ensure cryoprotection. On the next day, hemispheres were frozen in liquid isopentane and stored at -80°C until cutting.

### Antibodies

N-terminal antibody against seizure protein 6 (Sez6) was described previously [10, 21]. For detection of seizure 6-like protein (Sez6L) we used monoclonal antibody 21D9 [19]. Additionally, immunoblotting of mouse brain tissue lysates was performed using the following antibodies: anti-BACE1 (D10E5; Cell Signaling); anti-APP (22C11; Merck Millipore) for flAPP and sAPPt; anti-sAPPβ (SIG39138; Covance) and anti-actin (Sigma Aldrich). For immunohistochemistry of mouse brain tissue, the following antibodies were used: anti-BACE1 (Epitomics; Abcam), anti-APP (Y188; Epitomics, Abcam), anti-GFAP (Dako), anti-CD45 (BD Biosciences) and anti-calbindin (Swant). Immunocytochemistry of primary mouse cortical neurons was performed using these antibodies: anti-BACE1 (Epitomics; Abcam), anti-EEA1 (Cell Signaling), anti-transferrin receptor (TfR; ThermoFisher Scientific) and anti-LAMP1 (1D4B, Santa Cruz Biotechnology).

### Preparation of mouse brain tissue lysates and immunoblotting

Preparation of mouse brain tissue lysates for analysing BACE1 proteolysis of its substrates was performed as in Kuhn et al. [10]. Dissected tissues from NPC1^+/+^ (wt) and NPC1^-/-^ (NPC1) mice were weighed and ten-fold volume to weight ratio of homogenization buffer was added for homogenization of each tissue sample, as follows: to generate soluble fractions, tissues were homogenized in 0.25% DEA, 100mM NaCl buffer, containing protease inhibitor cocktail (Complete, Roche Applied Science), in a glass homogenizer until tissue lysate appeared uniform. The centrifugation was carried out at 100 000 × g at 4°C for 30 minutes. After the centrifugation, tissue supernatants were collected, placed in fresh tubes and stored at -80°C freezer until further use. To generate membrane-bound fractions, the obtained tissue pellets were further homogenized in the same volume of 1% Triton buffer [1% Triton X-100, 150mM NaCl, 50mM Tris-HCl, pH 7.4 and 2mM EDTA]—containing protease inhibitor cocktail (Complete, Roche Applied Science) by using a glass homogenizer. The lysates were then pressed through a 23-gauge needle by using a 1 ml syringe until tissue lysates appeared uniform. These tissue lysates were left on ice for 30 minute incubation. Following this step, the lysates were centrifuged at 100 000 × g at 4°C for 30 minutes. After the centrifugation, tissue supernatants were collected, placed in fresh tubes and stored at -80°C freezer until further use. Proteins of interest were detected using SDS-PAGE/Western blotting, under reducing and non-reducing conditions (for Sez6L) on Tris-Glycine gels, followed by immunoblotting with specific antibodies.

### Analyses of protein levels in mouse brain tissue lysates

For analyses of different proteins, six NPC1^+/+^ (wt) and six NPC1^-/-^ (NPC1) mice (mixed-sex) were used at both 4- and 10-weeks of age. For analyses of proteins in specific brain regions, all mouse brain protein lysates were separated by using one 15-well SDS-PAGE gel. In addition, a protein standard, represented by either one mouse brain-DEA- or one mouse brain-1% Triton-buffer lysate, was loaded in the end two lanes of every gel, to allow comparison of results between gels and experiments. Protein signals were quantified by using the ImageJ software (National Institutes of Health, USA). Signal values obtained for all proteins of interest were divided with an average signal value of the corresponding protein standards loaded on each gel. Then, each protein signal was normalized against actin values (either “Actin-DEA” for DEA lysates or “Actin-Triton” for 1% Triton buffer lysates) to correct for protein content. Comparison of protein levels between two groups of data (wt- vs. NPC1-mice) was performed by using a two-tailed, unpaired *t*-test. Differences were considered significant at a *p* value of <0.05 (* <0.05; ** <0.005).

### Immunohistochemistry of mouse brain cryosections

The immersion-fixed and cryprotected frozen right hemispheres from wt and NPC1 mice were used to prepare 10 μm thick sagittal cryosections for histological analysis on a Leica CM 3050S cryotome. Sections were stored at -20°C until used for immunohistochemistry.

For immunohistochemistry, cryosections were briefly washed in PBS containing 0.5% Triton X-100 (PBS-T) and blocked in 5% goat serum in PBS-T for 1 hour. Sections were subsequently incubated with primary antibodies diluted in 5% goat serum in PBS-T overnight. After 3–5 hours incubation with secondary antibody (conjugated to Alexa Fluor 488, Thermo Fisher Scientific), DAPI (Sigma Aldrich) was used to counterstain nuclei. Images were acquired on a Zeiss AX10 fluorescent microscope using the software AxioVision Release 4.8 (Zeiss).

### Immunocytochemistry of mouse primary cortical neurons

Cortical neurons were isolated from postnatal day 0 (P0) mouse pups and grown as described previously [24]. Neurons were plated on poly-L-lysine (0.5 mg/mL, Sigma Aldrich) coated glass coverslips (Marienfeld) and grown in Neurobasal medium complemented with 2% B27 and 2mM L-glutamine (all from Gibco, Thermo Fisher Scientific). Immunocytochemistry on cortical mouse neurons was performed as previously described [14]. In short, cortical neurons grown on coverslips were fixed with 4% sucrose/paraformaldehyde for 15 minutes, quenched in 50mM ammonium chloride and permeabilized with 0.1% Triton X-100 for 3 minutes. The neurons were blocked at room temperature for 1 hour in 5% goat serum (Sigma Aldrich). Neurons were subsequently incubated with primary antibodies diluted in blocking solution overnight, followed by secondary antibody incubation (Alexa Fluor 488, 594 and 647, Thermo Fisher Scientific) together with filipin (Sigma Aldrich, as described by Neufeld et al. 1999). Confocal images were acquired on an inverted laser scanning confocal microscope Leica TCS SCP8. Images were acquired with the software LAS X (Leica) and additional image processing and were performed using ImageJ software (National Institutes of Health, USA).

## Results

### β-Secretase processing of Sez6, Sez6L and APP in NPC1 mouse brains

We firstly analysed protein levels of Sez6 and Sez6L, as well as APP, in wt and NPC1 mouse brains. For this, we monitored soluble and membrane-bound metabolites of Sez6, Sez6L and APP in cortex, hippocampus and cerebellum of 4- and 10-week old NPC1 mice compared to wt mice. The age of mice was selected based on the previously documented progression of the NPC disease in this mouse model (NPC1 mice usually die between 10–14 weeks of age) [25, 26]. While 4-weeks old NPC1 mice seem to be unaffected, at 10-weeks there is a profound neurodegeneration, in particular of Purkinje neurons of the cerebellum (S1 Fig), a characteristic feature of disease progression [25, 26].

**Fig 1:**
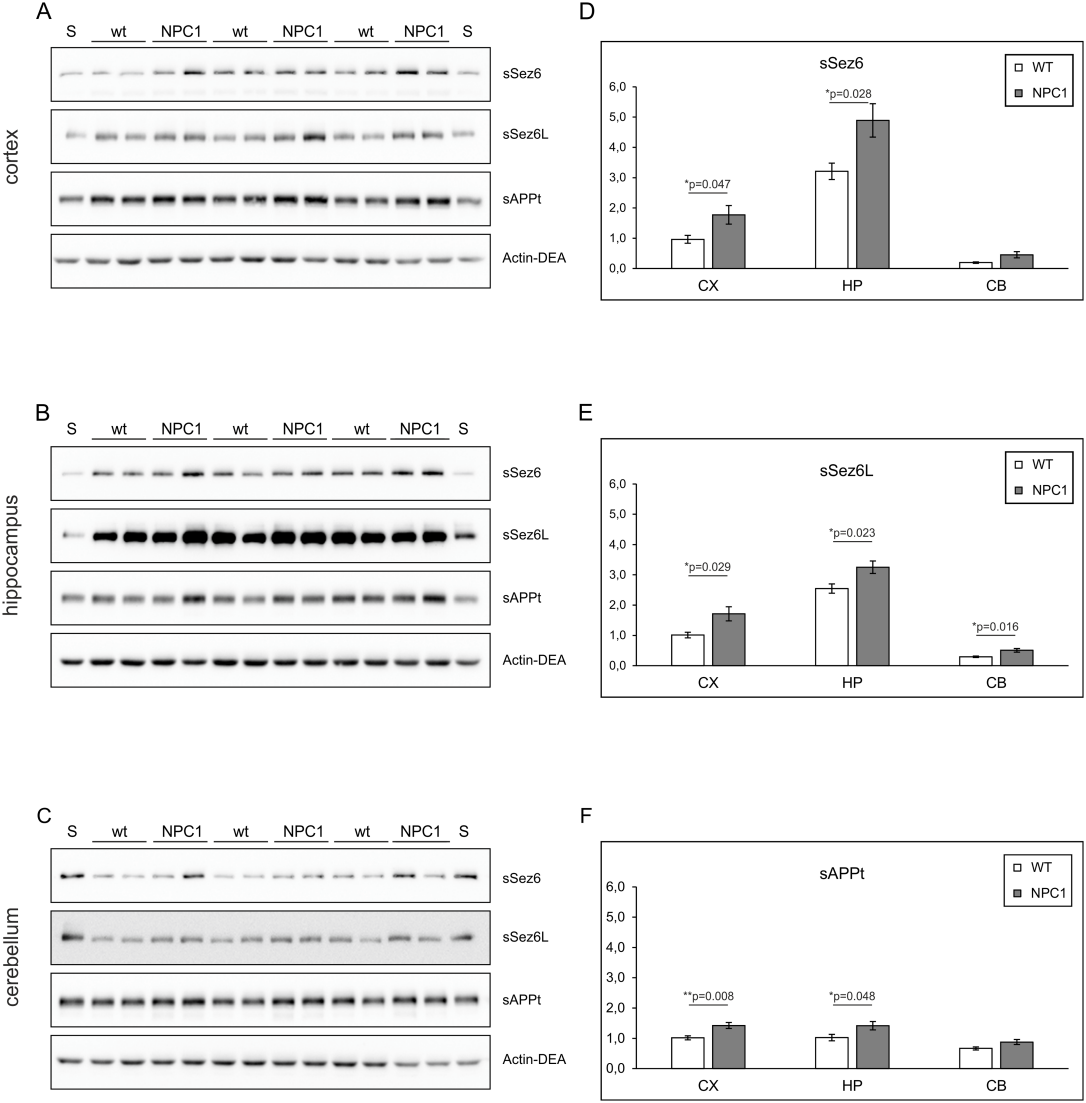
The increased levels of soluble Sez6, Sez6L and APP ectodomains reveal enhanced proteolysis by BACE1 in 4-weeks old NPC1 vs. wt mouse brains. (A-C) Western blot analyses of soluble Sez6 (sSez6), soluble Sez6L (sSez6L), total soluble APP (sAPPt) and actin (Actin-DEA) in DEA fractions of the cortex (A), hippocampus (B) and cerebellum (C) collected from 4-weeks old wt (NPC1^+/+^; N = 6) and NPC1 (NPC1^-/-^; N = 6) mice. A standard protein lysate (S) was included twice on each gel. (D-F) Graphs representing quantified protein signals of sSez6 (D), sSez6L (E) and sAPPt (F) which were normalized against actin (Actin-DEA) in the cortex (CX), hippocampus (HP) and cerebellum (CB) of 4-weeks old wt- and NPC1-mice.

To analyse the soluble fragments and the full-length or membrane-bound proteins, mouse brains were sequentially homogenized in DEA (soluble fractions) and Triton X-100 (membrane-bound fractions) homogenization buffers [10]. In 4-weeks old NPC1 vs. wt mice, significantly increased levels of the soluble Sez6 (sSez6) and soluble Sez6L (sSez6L), as well as of total, soluble APP levels (sAPPt) were detected in the cortex and hippocampus (Fig 1A, 1B and 1D–1F). In the cerebellum at 4-weeks of age, however, only sSez6L levels were significantly enhanced in the NPC1 mice compared to the wt mice (Fig 1C and 1E). Although the levels of sSez6 and sAPPt seem to increase in cerebellum of NPC1 compared to wt mice, this did not reach statistical significance (Fig 1C, 1D and 1F). At this age, no changes in the levels of the corresponding full-length proteins Sez6, Sez6L and APP, as well as of BACE1, were detected in these brain regions (S2 Fig).

**Fig 2:**
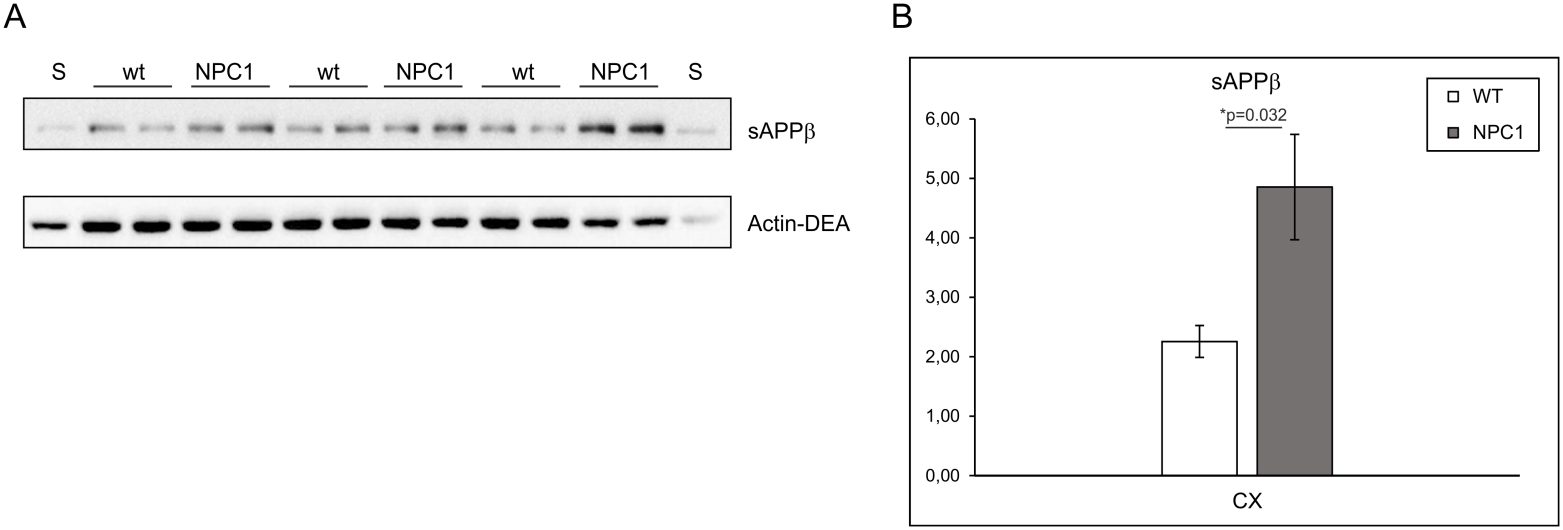
The levels of BACE1-generated soluble APPβ fragments are increased in the cortex of 4-weeks old NPC1 vs. wt mice. (A) Western blot analysis of soluble APPβ (sAPPβ) and actin (Actin-DEA) protein levels in DEA fractions of the cortex collected from 4-weeks old wt (NPC1^+/+^; N = 6) and NPC1 (NPC1^-/-^; N = 6) mice. A standard protein lysate (S) was included twice on each gel. (B) A graph representing quantified protein signals for soluble APPβ (sAPPβ) in the cortex of 4-weeks old animals which were normalized against actin protein levels (Actin-DEA).

We also analysed the levels of sAPPβ, as sAPPβ is a direct product of APP processing by BACE1 alone, while sAPPt fragment results from both BACE1 and an α-secretase cleavage of APP. Significantly increased sAPPβ levels were detected in the cortex of the 4-weeks old NPC1 vs. wt mice (Fig 2), confirming an enhanced β-secretase processing of APP in the cortex of 4-weeks old NPC1 mice. We were unable to detect sAPPβ in the lysates of hippocampus and cerebellum of these mice.

**Fig 3:**
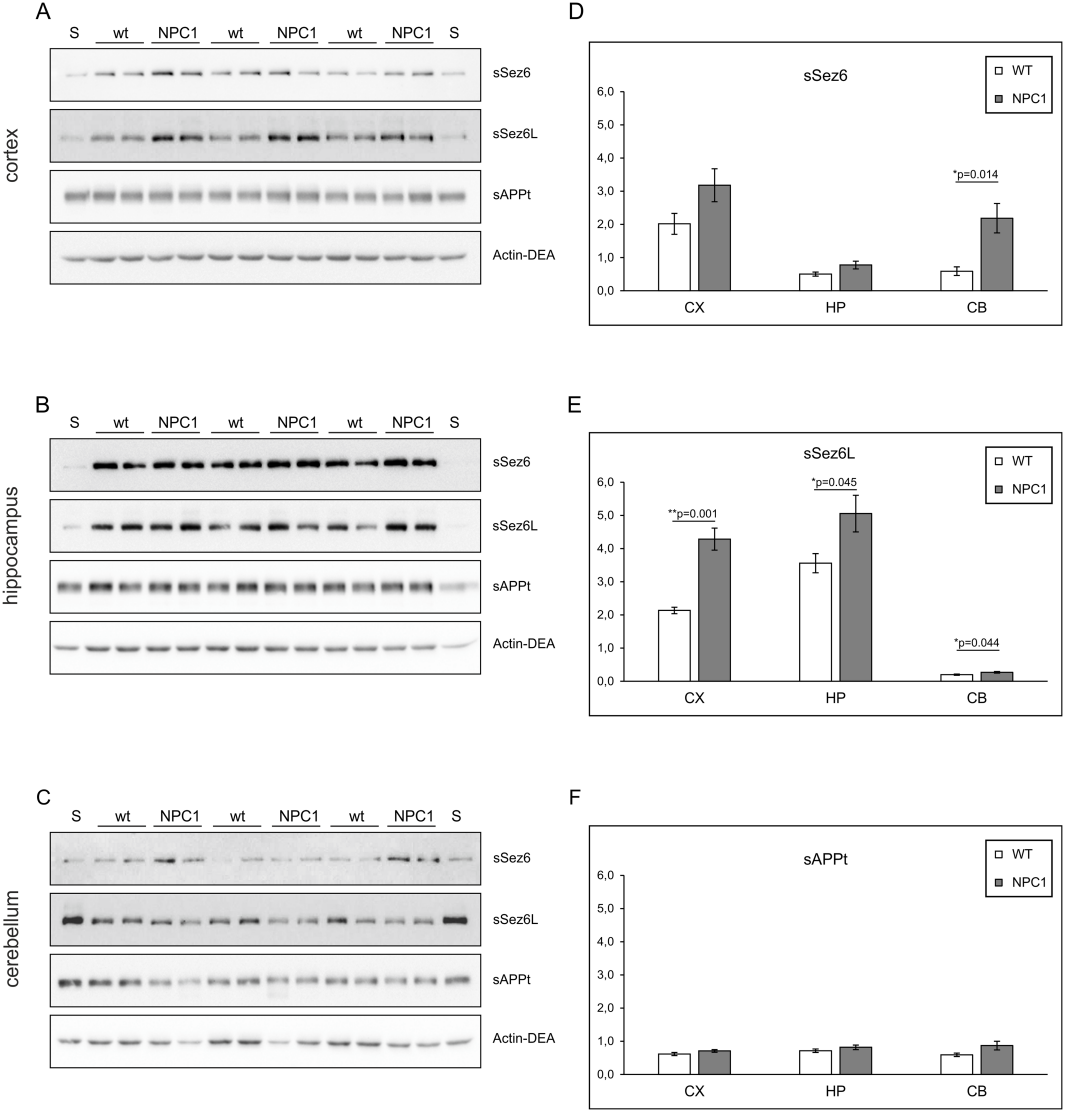
At 10-weeks of age an enhanced BACE1-mediated proteolysis of Sez6L is present in all three brain regions of NPC1 vs. wt mice, while cleaved Sez6 is increased only in the cerebellum. (A-C) Western blot analyses of soluble Sez6 (sSez6), soluble Sez6L (sSez6L), total soluble APP (sAPPt) and actin (Actin-DEA) in DEA fractions of the cortex (A), hippocampus (B) and cerebellum (C) collected from 10-weeks old wt (NPC1^+/+^; N = 6) and NPC1 (NPC1^-/-^; N = 6) mice. A standard protein lysate (S) was included twice on each gel. (D-F) Graphs representing quantified protein signals of sSez6 (D), sSez6L (E) and sAPPt (F) which were normalized against actin (Actin-DEA) in the cortex (CX), hippocampus (HP) and cerebellum (CB) of 10-weeks old wt (NPC1^+/+^; N = 6) and NPC1 (NPC1^-/-^; N = 6) mice.

At 10-weeks of age statistically significant changes in the levels of sSez6L were detected in all three brain regions analysed in NPC1 vs. wt mice (Fig 3A–3C and 3E). For sSez6, however, significantly increased levels were observed only in the cerebellum of NPC1 mice compared to wt ones (Fig 3C and 3D). No statistically significant changes in the levels of the full-length proteins Sez6, Sez6L, APP, as well as of BACE1, were detected at this age (S3 Fig). We were unable to detect sAPPβ fragment in mouse brain tissue lysates of mice at 10-weeks of age.

When comparing BACE1-mediated proteolysis of all three substrates between 4- and 10-weeks of age, we noticed an interestingly opposing trend of BACE1-proteolysis between Sez6 and Sez6L in the hippocampus and cerebellum of wt and NPC1 mice. While the levels of sSez6 in hippocampus substantially decreased by age (by nearly 4-fold), in the cerebellum they were markedly increased by nearly 5-fold (Figs 3D vs. 1D). In the cortex sSez6 levels were further increased with age (by almost 2-fold). Interestingly, significantly enhanced BACE1-cleavage of Sez6L persisted in all three brain regions in NPC1 mice at both 4- and 10-weeks of age (Figs 1E and 3E).

### APP and BACE1 distribution in NPC1 mouse brains revisited

**Fig 4:**
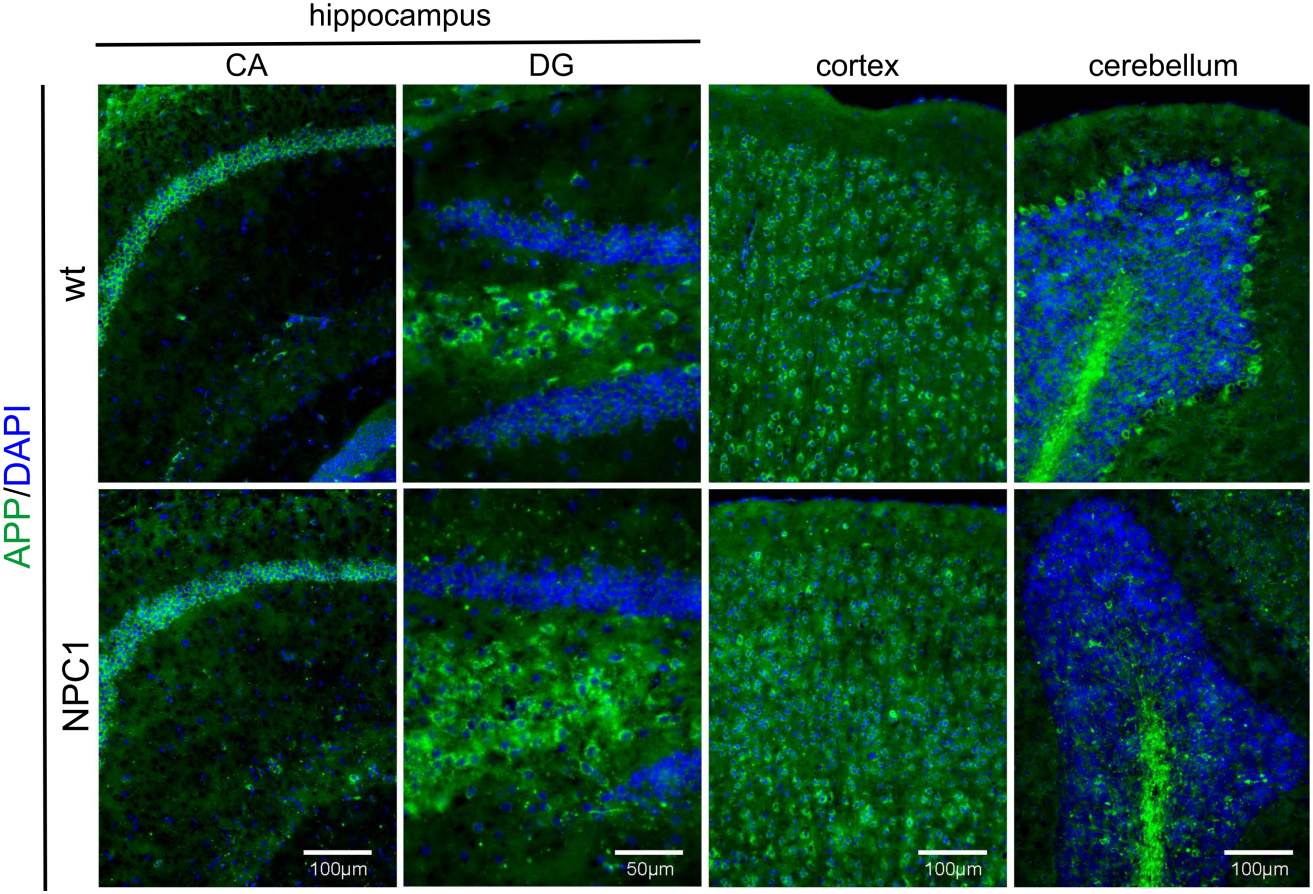
APP immunoreactivity is lost in Purkinje neurons upon their neurodegeneration in 10-weeks old NPC1 vs. wt mouse brains. APP (green) is expressed in neurons of the cornu ammonis (CA) regions and dentate gyrus (DG) of the hippocampus, as well as in cortical neurons and Purkinje cells in the cerebellum in 10-weeks old wt mice. Due to profound neurodegeneration, 10-weeks old NPC1 mice show a substantial loss of APP intensity in Purkinje neurons. DAPI (blue) was used to counterstain all nuclei.

**Fig 5:**
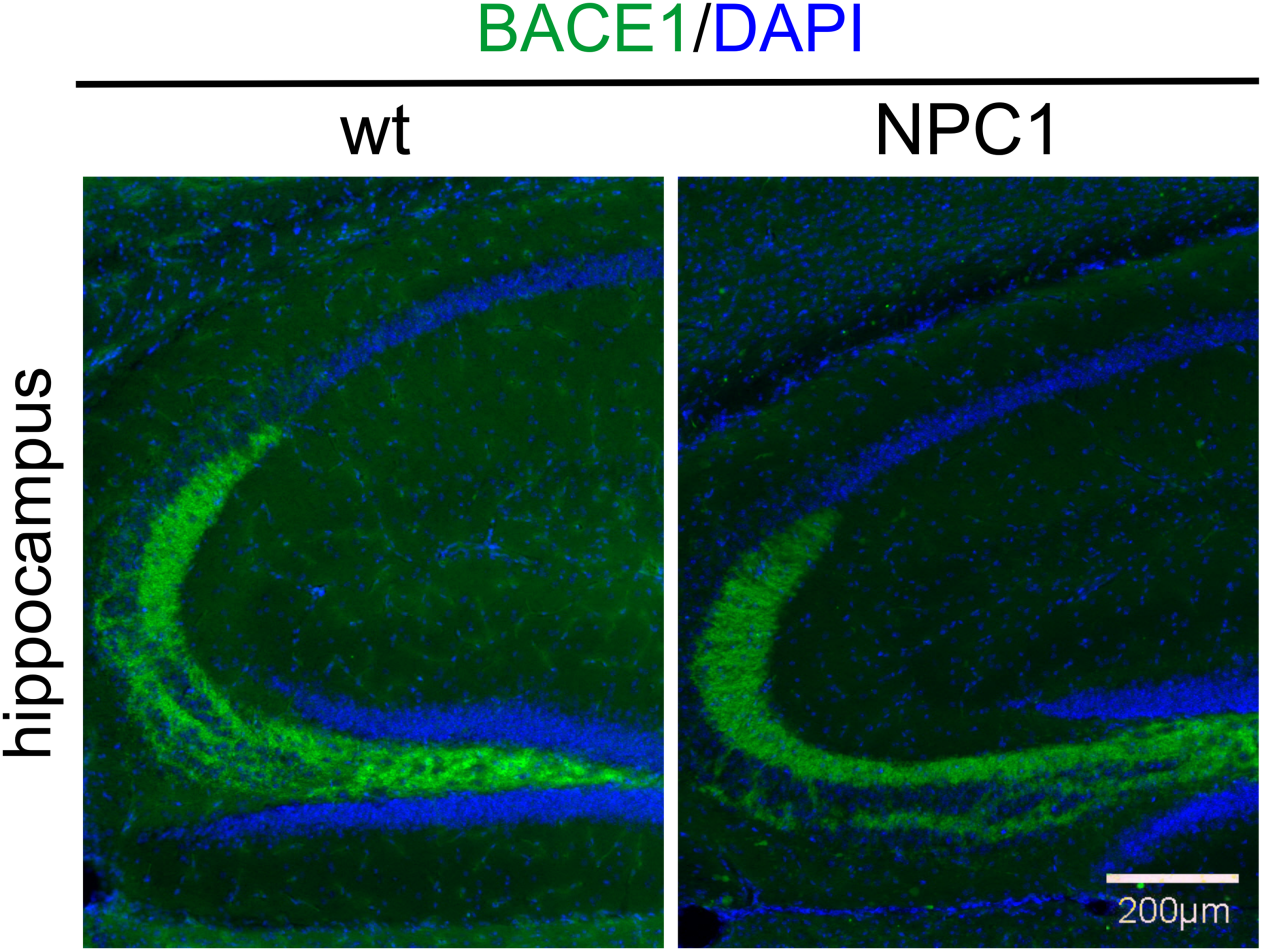
BACE1 distribution is similar between 10-weeks old NPC1 vs. wt mouse brains. BACE1 (green) is intensely staining mossy fibers in the hippocampus of both wt and NPC1 mice. DAPI (blue) was used to counterstain all nuclei. There may be a slight decrease of BACE1 immunoreactivity in NPC1 mouse brains most likely due to profound neuropathological processes at this stage.

Previous work by Kodam et al. [27] showed that in NPC1 mouse brains APP intensely stained hippocampal neurons of the cornu ammonis regions 1–3 (CA1-3) and dentate gyrus (DG) as well as Purkinje neurons in the cerebellum. Interestingly, they also reported increased APP immunoreactivity in astrocytes in the hippocampus and cerebellum of 4-, 7- and 10-weeks old NPC1 mice. The reported false positive immunostaining of several widely used APP antibodies and the high specificity of a rabbit monoclonal antibody Y188 [28] prompted us to re-examine the distribution of endogenous APP in wt and in NPC1 mouse brains, respectively. In contrast to earlier study [27], we detected APP immunoreactivity specifically in neurons. Accordingly, we observed an intense staining of APP in hippocampal neurons (specifically pyramidal neurons of CA1, neurons in the hilus and granular neurons of DG), cortical neurons and in Purkinje neurons of the cerebellum (Fig 4). Also, similar distribution and levels of APP were observed between wt and NPC1 mice at both 4- (not shown) and 10-weeks of age, except that, due to profound neurodegeneration of Purkinje neurons in 10-weeks old NPC1 mice (S1 Fig) a substantial loss of APP intensity in Purkinje neurons was detected (Fig 4). Additionally, APP immunoreactivity did not resemble the enhanced staining pattern of astrocytes in 10-weeks old NPC1 mice (S4 Fig). The immunostaining of BACE1 revealed that there is no increased BACE1 staining in 4- (not shown) and 10-weeks old NPC1 vs. wt mouse brains that would be of relevance for the enhanced BACE1-proteolysis (Fig 5). BACE1 intensely stained mossy fibers in the hippocampus (Fig 5) as also recently reported by Kandalepas et al. [29]. There may be a slight decrease of BACE1 immunoreactivity in NPC1 mouse brains at 10-weeks of age most likely due to neuropathological processes at this stage. The specificity of the BACE1 antibody used was verified in BACE1 KO mouse brain slices which did not show any BACE1 immunoreactivity (S5 Fig).

### Sez6 and Sez6L distribution in NPC1 mouse brains

**Fig 6:**
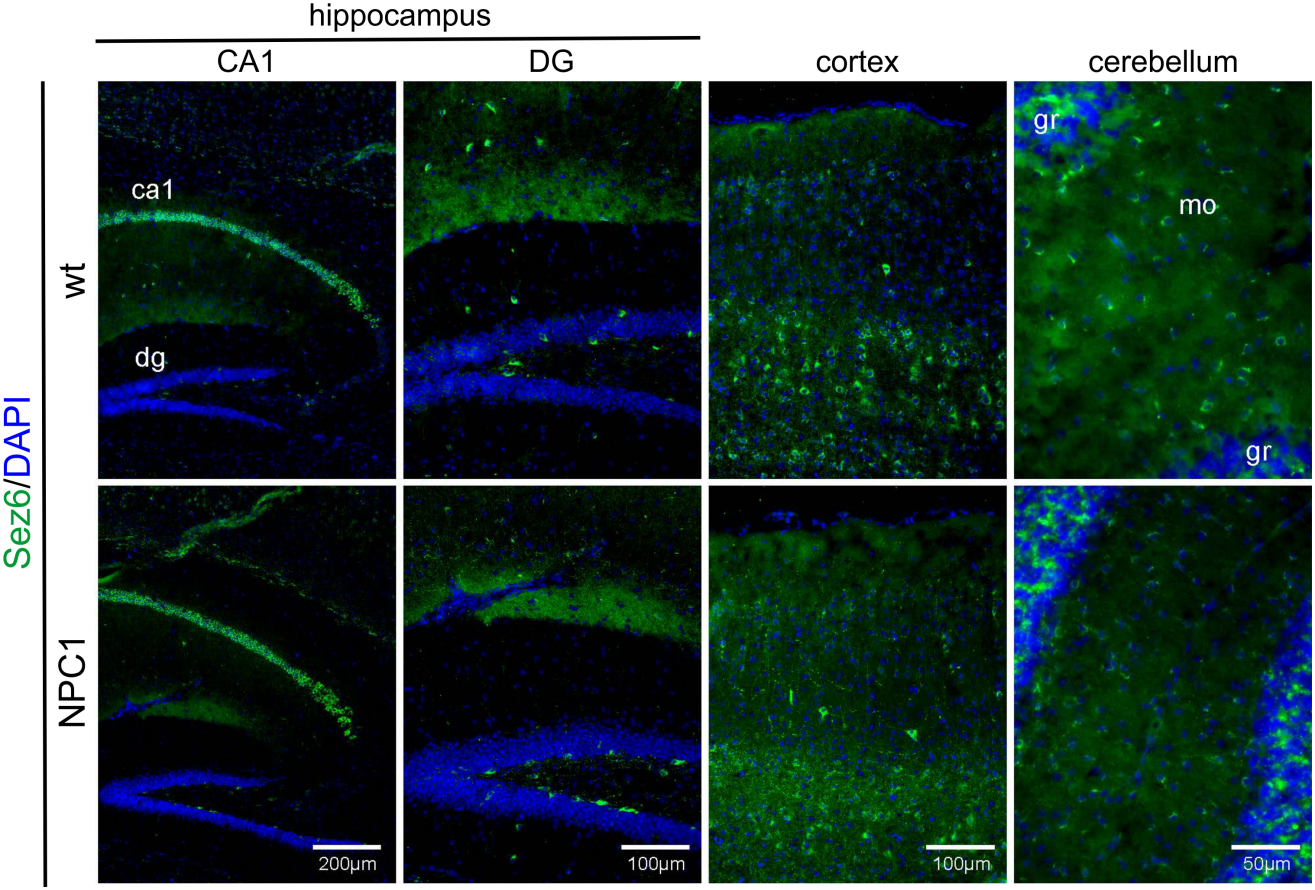
Sez6 distribution is similar in the brains of 10-weeks old NPC1 and wt mouse. Sez6 (green) is specifically expressed in neurons of the region cornu ammonis 1 (CA1) and hilus of dentate gyrus (DG) of the hippocampus, as well as in deep layer cortical neurons and neurons in the molecular layer of the cerebellum in both 10-weeks old wt and NPC1 mouse brains. DAPI (blue) was used to counterstain all nuclei. ca1, cornu ammonis 1; dg, dentate gyrus; mo, molecular layer; gr, granular layer.

**Fig 7:**
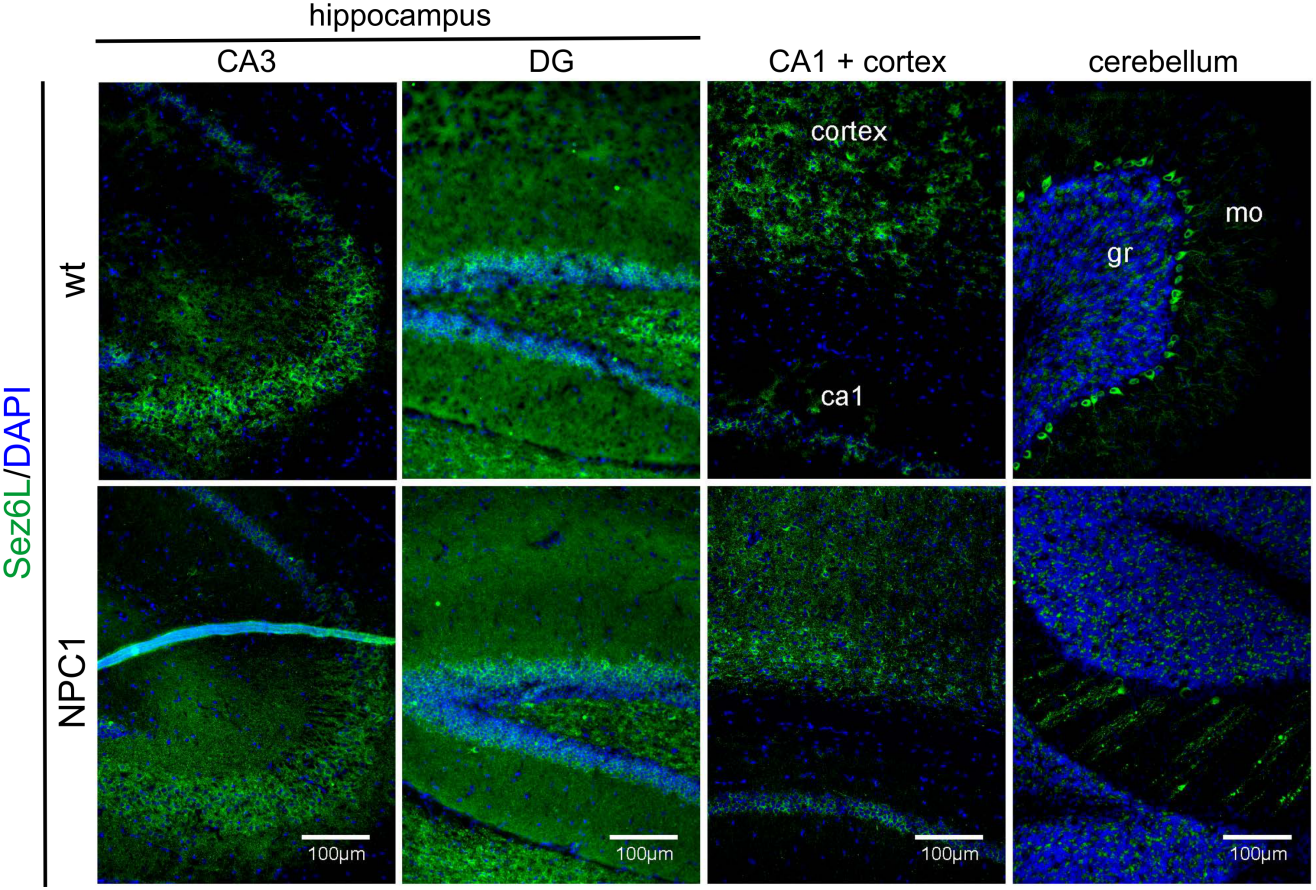
Sez6L immunoreactivity is lost in Purkinje neurons upon their neurodegeneration in 10-weeks old NPC1 vs. wt mouse brains. Sez6L (green) is specifically expressed in neurons of the cornu ammonis (CA) and granular layer of dentate gyrus (DG) of the hippocampus, as well as in deep layer cortical neurons and Purkinje cells in the cerebellum in 10-weeks old wt mice. Due to profound neurodegeneration, 10-weeks old NPC1 mice show a substantial loss of Sez6L intensity in Purkinje neurons. DAPI (blue) was used to counterstain all nuclei. ca1, cornu ammonis 1; mo, molecular layer; gr, granular layer.

Next, we performed immunohistochemistry of Sez6 and Sez6L in brain sections of 4- (not shown) and 10-week old NPC1 mice vs. wt mice. Both Sez6 and Sez6L showed a preferential neuronal-like staining (Figs 6 and 7, respectively). Additionally, Sez6/Sez6L immunoreactivity did not resemble the enhanced staining pattern of astrocytes and microglia in 10-weeks old NPC1-mice (S4 and S6 Figs, respectively), indicating that these proteins are most likely not expressed in glial cells analysed. Sez6 showed strong neuronal-like cell body immunoreactivity in the hippocampal CA1 region, some in the hilus of DG and in deep layers of the cortex, as previously described by Gunnersen et al. [21] and by Osaki et al. [22]. Also, we detected Sez6 cell body immunoreactivity in the cerebellar molecular layer in 10-weeks old wt mice (Fig 6). We observed no changes of Sez6 levels and distribution in 4- (not shown) and 10-weeks old NPC1 vs. wt mice. Similarly, neuronal-like immunoreactivity of Sez6L was detected in the hippocampal CA regions, granular neurons of DG as well as in deep layers of cortex (Fig 7). Immunostaining of Sez6L in the hippocampus and cortex of 10-weeks old NPC1 mice did not differ from that in the wt mice. Interestingly, in 4- (not shown) and 10-weeks old wt mice Sez6L showed a strong immunoreactivity in Purkinje neurons of the cerebellum which was lost upon Purkinje cell neurodegeneration in NPC1 mice (Fig 7).

### Subcellular distribution of Sez6, Sez6L and BACE1 in NPC1 primary mouse cortical neurons

**Fig 8:**
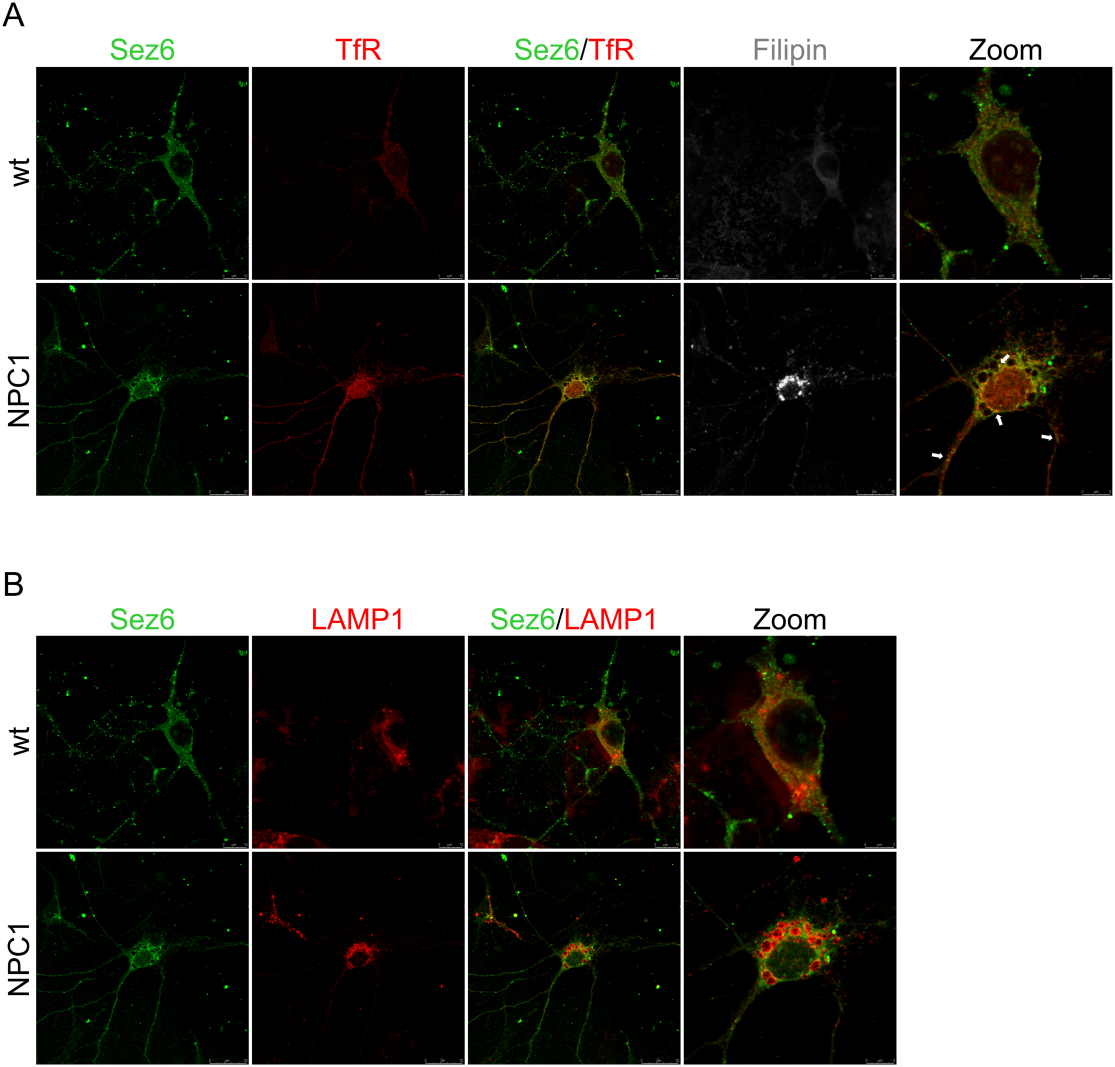
NPC1 mouse primary cortical neurons show increased punctuate staining of Sez6 within endosomal compartments. Representative images of primary mouse cortical neurons stained with Sez6 antibody and endolysosomal markers: transferrin receptor (TfR) and lysosomal-associated membrane protein 1 (LAMP1). Note that Sez6 in NPC1^+/+^ (wt) cortical neurons was localized in the soma and in neuronal processes. Upon NPC1-loss and cholesterol accumulation in NPC1^-/-^ (NPC1) cortical neurons, its immunoreactivity showed more punctate staining in the soma (in vesicles that do not accumulate cholesterol) and in the processes (indicated by arrows).

**Fig 9:**
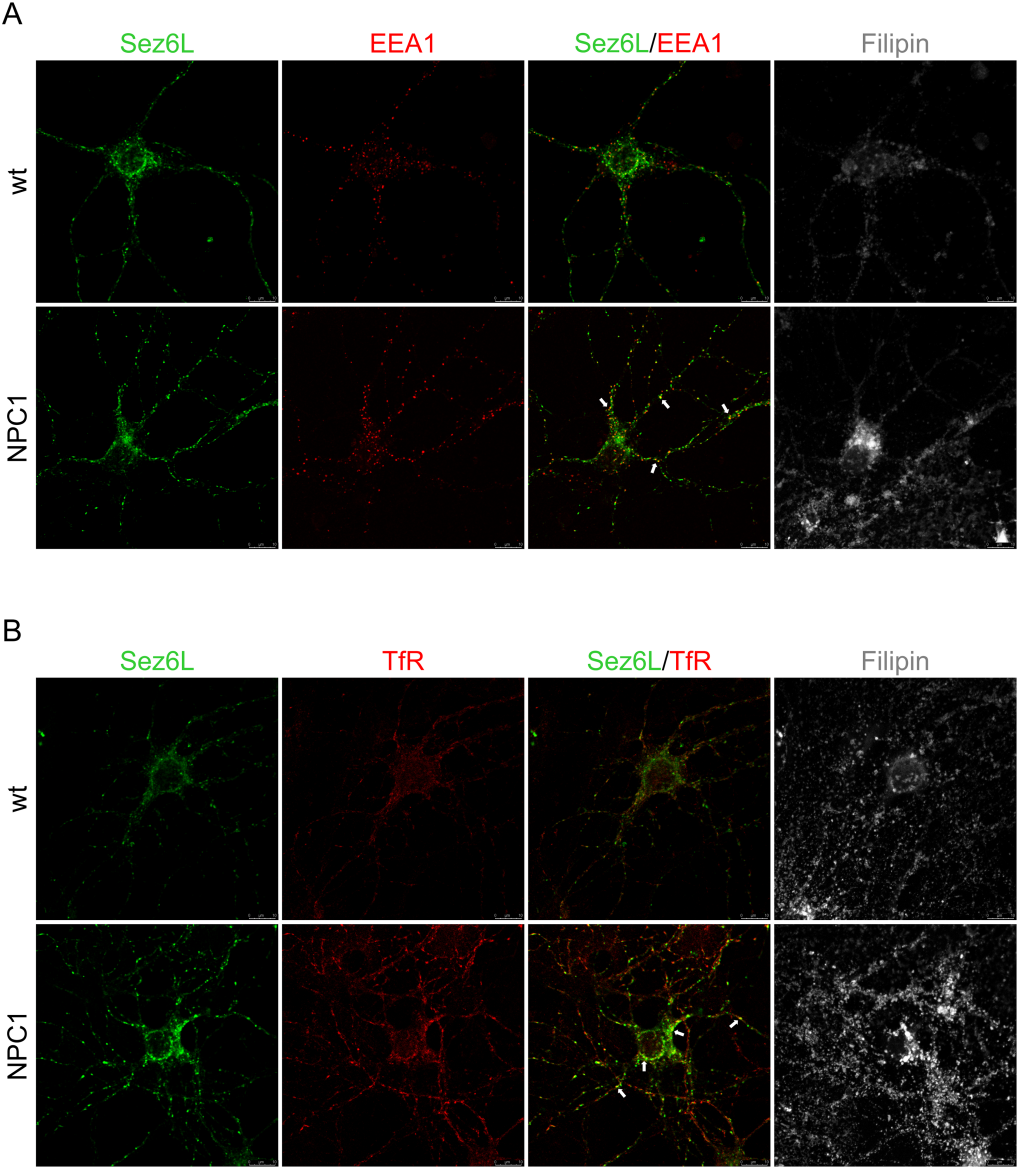
NPC1 mouse primary cortical neurons show profound Sez6L punctuate staining in neuronal processes. Representative images of primary mouse cortical neurons stained with Sez6L antibody and endocytic markers: early endosome antigen (EEA1) and transferrin receptor (TfR). Immunoreactivity of Sez6L in NPC1^+/+^ (wt) cortical neurons was detected in both soma and neuronal processes. Upon NPC1-loss and cholesterol accumulation in NPC1^-/-^ (NPC1) cortical neurons Sez6L showed more enlarged punctate staining mainly in TfR-positive vesicles in the soma and in neuronal processes (indicated by arrows).

**Fig 10:**
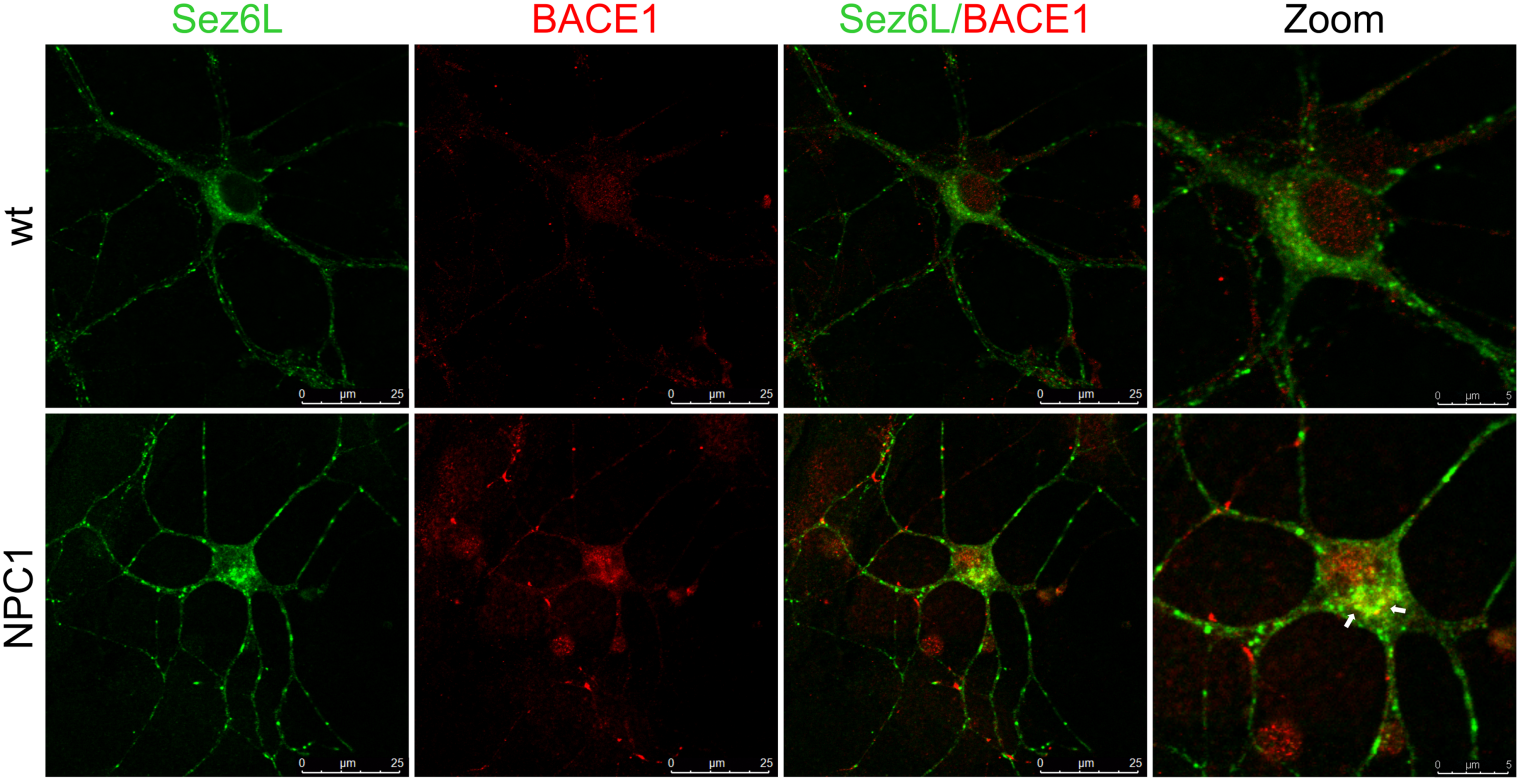
Sez6L co-staining with BACE1 reveals their increased immunoreactivity in the cell body of NPC1 vs. wt mouse primary cortical neurons. Representative images of primary mouse cortical neurons stained with Sez6L antibody co-stained with BACE1. In NPC1^-/-^ (NPC1) cortical neurons, in contrast to NPC1^+/+^ (wt) neurons, we observed an increased punctate staining of Sez6L in the cell body and in the neuronal processes. The cell body co-staining of Sez6L and BACE1 was enhanced in NPC1 vs. wt neurons (indicated by arrows).

In a previous study we showed that both NPC1-loss and NPC1-dysfunction, due to U18666A-treatment, caused sequestration of APP and BACE1 in acidic early/recycling endocytic compartments [14] in which BACE1 is the most active. We assumed that accumulation of the substrate APP and its cleaving enzyme BACE1 in this optimal subcellular environment causes enhanced β-secretase cleavage of APP in NPC disease, which we have previously described in Malnar et al. [30]. To test whether similar trafficking defect of BACE1 and its substrates, Sez6 and Sez6L, could be observed upon cholesterol accumulation due to NPC1 loss-of-function, we monitored the subcellular localization of BACE1 and Sez6 or Sez6L, in primary mouse cortical neurons isolated from NPC1^-/-^ (NPC1) and NPC1^+/+^ (wt) mice at postnatal day 0 (P0). NPC1 cortical neurons showed accumulation of free cholesterol in the cell body, as shown by filipin staining, and enlarged lysosomes (LAMP1)—characteristic features of NPC disease (Figs 8 and 9). The endo/lysosomal markers of early endosomes (EEA1) and recycling endosomes (TfR) (Figs 8 and 9) showed altered staining in NPC1 mouse primary cortical neurons, likely reflecting a cholesterol-mediated defect within the endosomal/lysosomal compartment. We noticed more puncta of EEA1 and TfR in the processes of NPC1 cortical vs. wt neurons (Figs 8 and 9). Sez6 and Sez6L in wt neurons were localized in the soma and in the neuronal processes (Figs 8 and 9). Interestingly, in the NPC1 neurons their localization was more pronounced in the neuronal processes, especially for Sez6L (Figs 8 and 9). Upon NPC1-loss, Sez6 and Sez6L showed no colocalization with cholesterol-rich late endosomes/lysosomes (stained with LAMP1 and filipin). Instead, we observed enhanced punctuate staining of both Sez6 and Sez6L with TfR in the soma as well in the neuronal processes of NPC1 vs. wt cortical neurons (Figs 8A and 9B). When Sez6L was co-stained with BACE1 we detected their increased immunoreactivity in the cell body of NPC1 vs. wt cortical neurons (Fig 10). Unfortunately, since both Sez6 and BACE1 antibodies were raised in rabbit we could not analyse their colocalization in mouse primary cortical neurons. Altogether, these results demonstrate that cholesterol accumulation due to NPC1-dysfunction causes altered trafficking of BACE1 substrates Sez6 and Sez6L, within the endolysosomal pathway.

## Discussion

The protease BACE1 is currently the most promising drug target for developing treatments against AD [18, 31]. The recently identified APP variant Ala673Thr, which is less efficiently cleaved by BACE1 [32], together with studies on AD-transgenic mice crossed with genetically deleted BACE1 mice, showing a decrease in Aβ levels and protection against AD and cognitive decline [33, 34, 35, 36, 37], strongly suggest that BACE1-inhibition should prove effective for AD treatment. However, recent studies on BACE1^-/-^ mice have shown complex neurological phenotypes, raising a concern that a complete BACE1-inhibition might not be as safe as initially expected [38]. Indeed, recently several clinical trials were stopped because of unacceptable side-effects [39]. Such side-effects may result from a reduced cleavage of other physiologically relevant BACE1 substrates, as BACE1 has additional functions in the brain besides cleaving APP [40, 41]. Thus, it is important to fully understand the basic biology of BACE1 and the effects of the cleavage of its substrates to better evaluate the therapeutic potential of BACE1 and make clinical trials safer.

In this work, we employed a single gene lysosomal disorder of cholesterol accumulation—NPC, which was previously shown to share several similarities with complex AD, to further characterize proteolysis carried out by BACE1. NPC disease is also referred to as “juvenile Alzheimer’s disease” and is characterized by progressive neurodegeneration causing symptoms which include ataxia, dystonia, seizures and dementia. Here, we show for the first time that BACE1-dependent proteolytic cleavage is enhanced in a mouse model of NPC disease suggesting that upregulation of BACE1-dependent cleavage could represent an additional common feature between NPC disease and AD. To characterize BACE1 proteolysis in NPC disease, we tested Sez6 and Sez6L, which were shown to be predominantly cleaved by BACE1 in primary cultured neurons [10, 19]. In parallel, we analysed proteolysis of APP which is, besides BACE1, also cleaved by another protease—ADAM10 [18, 20]. In agreement with our previous result of increased amyloidogenic APP processing by BACE1 in the NPC1-null cellular model [30], here, we describe increased proteolysis of the BACE1 substrates Sez6 and its homolog Sez6L, as well as APP, in the cortex and the hippocampus of NPC1 mice at 4-weeks of age while, in the cerebellum, increased BACE1 proteolysis was prominent only for Sez6L protein. The detected upregulation of Sez6, Sez6L and APP cleavage by BACE1 in NPC1 mouse brains, at 4-weeks of age, i.e. early stages in NPC disease pathogenesis [25, 26] is of particular relevance as it implies that enhanced BACE1-dependent cleavage is an early pathological event that precedes neurodegeneration and fully blown disease pathology. Increased BACE1 proteolysis could potentially be used as an early biomarker of NPC disease. It is worth to mention that the observed elevated BACE1 proteolysis of all three BACE1 substrates in nearly all three brain regions analysed (cortex, hippocampus and cerebellum) indicates that their enhanced BACE1 cleavage at this age is likely to be a general effect of NPC1 loss-of-function. However, upon neurodegeneration at 10-weeks of age [25, 26] increased BACE1-dependent proteolysis in NPC1 mice persisted only for Sez6L protein in all three brain regions analysed (it was even 2-fold increased in the cortex and hippocampus). Interestingly, Sez6 cleavage by BACE1 seemed to be strongly upregulated only in the cerebellum at this age, while it was substantially decreased in the hippocampus compared to the levels of cleaved Sez6 at 4-weeks of age. Although Sez6 and Sez6L belong to the family of Sez6 proteins, these results indicate that the efficacy of their cleavage by BACE1 is spatiotemporally differently regulated, particularly in the hippocampus and cerebellum (between 10- and 4-weeks of age), while it was similar in the cortex. At 10-weeks of age we also observed loss of immunoreactivity of APP and Sez6L in Purkinje neurons upon their degeneration in NPC1 vs. wt mice.

These results suggest that upregulation of soluble ectodomains of BACE1 substrates, such as Sez6 and Sez6L, could have important implications in NPC disease phenotype. Indeed, Gunnersen et al. [21] showed that secreted and membrane-bound isoforms of Sez6 exert opposing actions on neurite branching, indicating that secreted ectodomain of Sez6 (Sez6 isoform III) may act to enhance neurite number (to positively control dendritic branching) and, thus, influence synaptic connectivity. As synaptic dysfunction is an early pathological feature of both AD and NPC diseases, it remains to be determined whether BACE1 substrates, Sez6 and Sez6L, may play a role in NPC/AD disease pathogenesis. The common clinical features between these two disorders, such as dementia, presentation of seizures, dysarthria and dysphagia, indicate that both AD and NPC may share common molecular and/or cellular pathway(s) in the disease pathogenesis. A new data has recently been reported for the function of Sez6 protein in maintaining normal dendritic spine dynamics and synaptic structure and function, indicating that prolonged BACE1-inhibition impairs synaptic plasticity via Sez6 protein [12]. Since enhanced BACE1-proteolysis of Sez6L persisted in all three brain regions analysed at 4- and at 10-weeks of age of NPC1 mice, we reasoned that BACE1-inhibition may affect Sez6L function, in addition to Sez6 [12]. Further studies are, however, needed to clarify this in order to comprehend the consequence of prolonged BACE1-inhibition and its therapeutic potential against AD and, possibly, NPC disease.

This study is the first to provide data on spatiotemporal cleavage and distribution/localization of Sez6 and its homologue Sez6L in both wt and NPC1 mouse brains and primary cortical neurons. Distribution of Sez6L in the brains of wt and NPC1 mice was similar to that of Sez6 (mostly stained hippocampus and lower layers in the cortex), except for cerebellum which showed a strong Purkinje staining of Sez6L in wt-mice. Osaki et al. [22] have reported that immunoreactivity of Sez6 gradually decreased by age in the early postnatal period and in the adult forebrain (from P0-P14, and 10-months old mice), however, the strongest levels of Sez6 were detected in CA1 hippocampi, which is in accordance to our findings.

Due to neuropathological similarities between AD and NPC diseases, Kodam and colleagues previously examined protein levels and distribution of APP and its processing enzymes, β- and γ-secretase, in the same mouse model of NPC disease, as used in our study, and detected a statistically significant increase in BACE1 protein levels in NPC1 vs. wt mice in cerebellum at 7- and 10-weeks of age and in hippocampus at 10-weeks of age [27]. Although, in our study, we did not detect an increase in BACE1 protein levels in NPC1 vs. wt mice, our results demonstrate significantly increased processing of the exclusive BACE1 substrates Sez6 and Sez6L, as well as APP, in NPC1 mice, suggesting that BACE1-dependent cleavage in this mouse model of NPC disease is enhanced. Indeed, Kodam et al. [27] showed that activity of β-secretase (BACE1), but not that of α-secretase, was significantly increased both in the hippocampus and cerebellum of NPC1 mouse brains, supporting our findings.

Since it was recently reported that a number of antibodies used against APP are non-specific (as they showed immunoreactivity in APP KO brains), we re-examined APP distribution in NPC1 mice using the previously reported specific APP antibody Y188 (Epitomics, raised against the YENPTY motif at the C-terminal fragment of APP). Kodam et al. [27] also used APP antibody against C-terminal APP fragment (raised against 15 amino acids at the C-terminus followed by the c-Myc epitope) [42]. Our results of APP immunostaining in NPC1 vs. wt mouse brains using the Y188 APP antibody are similar to findings by Kodam et al. [27] showing a profound APP loss in Purkinje neurons at 10-weeks of age. APP was mainly detected in neurons, in accord with the earlier study by Guo et al. [28]. In contrast to Kodam et al. [27], we did not observe the increased levels of fl-APP in the hippocampus and cerebellum of 10-weeks old NPC1 vs. wt mice.

Furthermore, our results on NPC1 mouse primary cortical neurons show that a trafficking defect of BACE1 and its substrates Sez6 and Sez6L, within the endosomal compartments may explain the mechanistic details of enhanced cleavage by BACE1 in a mouse model of NPC disease. In line with our previous results on APP and BACE1 [14] we show that cholesterol accumulation in NPC1 vs. wt mouse primary cortical neurons causes sequestration of the enzyme BACE1 and its substrates Sez6 and Sez6L within the endolysosomal compartments. As these acidic organelles present an optimal environment for the enzyme BACE1 to cleave its substrates, we assume that, in addition to APP, Sez6 and Sez6L, and any other BACE1 substrate that is functionally linked to endolysosomal routing would display its enhanced proteolysis by BACE1 in NPC disease. Our immunocytochemistry results revealed somewhat different subcellular localization of Sez6 and its homologue Sez6L. While Sez6 was localized in the somatodendritic region in wt cortical neurons (in accord to the findings described by Gunnersen et al. [21] and by Osaki et al. [22]), Sez6L was mainly found in the soma and axons in wt cortical neurons and was distributed in larger puncta throughout the neuronal processes in NPC1 cortical neurons. The described different spatiotemporal BACE1-cleavage of Sez6 and Sez6L in NPC1-mouse brains in this work, implies that these BACE1 substrates, although being members of the same Sez6 protein family, may exert somewhat different but complementary functions.

In NPC disease, disturbed cholesterol trafficking and its accumulation within the late endosomes/lysosomes due to *NPC1*/*NPC2* dysfunction is the primary cause of the endolysosomal defect. Indeed, cholesterol-depletion by hydroxypropyl-β-cyclodextrin (HP-β-CD) showed beneficial effects in NPC1 mice [43, 44] and in a single NPC patient [45]. We propose that altered trafficking within the endolysosomal system caused by cholesterol accumulation may lead to some neuropathological features of NPC disease, including those mediated by altered BACE1-proteolysis of Sez6 and Sez6L proteins. Future studies are needed to better evaluate the potential role of Sez6 and Sez6L in NPC disease pathogenesis as well as to investigate the use of their enhanced BACE1-proteolysis as diagnostic and/or therapeutic biomarkers of NPC disease.

## Conclusions

In conclusion, we show here for the first time that NPC1 loss-of-function in a mouse model of NPC disease leads to enhanced cleavage of BACE1 substrates Sez6 and Sez6L, in addition to APP, and that this effect most likely involves altered trafficking of BACE1 and of its substrates within the endolysosomal system. In this work we also describe the spatiotemporal distribution and processing of Sez6 and Sez6L, members of Sez6 family, in wt and NPC1-mouse brains. We show that the efficacy of Sez6 cleavage by BACE1 may be spatiotemporally differently regulated, particularly in the hippocampus and cerebellum, in contrast to the cleavage of Sez6L. This study suggests that enhanced BACE1-regulated proteolysis may play a role in NPC disease pathogenesis. Both AD and NPC diseases are still untreatable disorders. Thus, understanding the basic biology of BACE1 and the effects of the cleavage of its substrates is important to better evaluate the therapeutic potential of BACE1.

## Supporting information

S1 Fig NPC1 mice show progressive neurodegeneration of cerebellar Purkinje neurons. Representative images of calbindin staining in 4-, 7- and 10-weeks old NPC1 mouse cerebellum. The NPC1 cerebella show a partial loss of Purkinje neurons at 7-weeks of age, while at 10-weeks the majority of Purkinje cell immunoreactivity is lost.
(TIF)

S2 Fig The levels of full-length Sez6, Sez6L, APP and BACE1 are similar between 4-weeks old NPC1 and wt mouse brains. (A-C) Western blot analyses of full-length Sez6 (flSez6), Sez6L (flSez6L), APP (flAPP), BACE1 and actin (Actin-TR) in 1% Triton X-100 (TR) fractions of the cortex, hippocampus and cerebellum collected from 4-weeks old wt (NPC1^+/+^; N = 6) and NPC1 (NPC1^-/-^; N = 6) mice. (D-G) Graphs representing quantified protein signals of flSez6 (D), flSez6L (E), flAPP (F) and BACE1 (G) which were normalized against actin (Actin-TR) in the cortex (CX), hippocampus (HP) and cerebellum (CB) of 4-weeks old animals.
(TIF)

S3 Fig The levels of full-length Sez6, Sez6L, APP and BACE1 are similar between 10-weeks old NPC1 and wt mouse brains. (A-C) Western blot analyses of full-length Sez6 (flSez6), Sez6L (flSez6L), APP (flAPP), BACE1 and actin (Actin-TR) in 1% Triton X-100 (TR) fractions of the cortex, hippocampus and cerebellum collected from 10-weeks old wt (NPC1^+/+^; N = 6) and NPC1 (NPC1^-/-^; N = 6) mice. (D-G) Graphs representing quantified protein signals of flSez6 (D), flSez6L (E), flAPP (F) and BACE1 (G) which were normalized against actin (Actin-TR) in the cortex (CX), hippocampus (HP) and cerebellum (CB) of 10-weeks old animals.
(TIF)

S4 Fig Astrogliosis in 10-weeks old NPC1 vs. wt mouse brains. Representative images of glial fibrillary acidic protein (GFAP) staining of cerebellum, cortex and hippocampus. NPC1 mouse brains show a strong immunoreactivity against GFAP indicating profound neuroinflammation, a characteristic feature of NPC disease.
(TIF)

S5 Fig Validation of BACE1 antibody in BACE1-null brains. The specificity of the BACE1 antibody (Epitomics, Abcam) was verified in BACE1^-/-^ mouse brain slices. We found BACE1 (green) specific staining only in the mossy fibers in the hippocampus of BACE1^+/+^ mice. DAPI (blue) was used to counterstain all nuclei.
(TIF)

S6 Fig Microglial activation in 10-weeks old NPC1 vs. wt mouse brains. Representative images of CD45 staining of cerebellum, cortex and hippocampus. NPC1 mouse brains show a strong immunoreactivity against CD45 indicating profound neuroinflammation, a characteristic feature of NPC disease.
(TIF)

## Abbreviations

AD: Alzheimer’s disease
ADAM: A Disintegrin and Metalloprotease
ADAM10: A Disintegrin and Metalloprotease metallopeptidase domain 10
APP KO: amyloid-β precursor protein knockout
APP: amyloid-β precursor protein
APP-CTFs: amyloid-β precursor protein C-terminal fragments
APPsw: amyloid-β precursor protein carring Swedish mutation (KM670/671NL)
APPwt: wild-type amyloid-β precursor protein
Aβ: amyloid-β peptide
Aβ42: amyloid-β peptide of 42 amino acids
BACE1: β-site amyloid precursor protein cleaving enzyme 1 (β-secretase)
CA1-3: cornu ammonis brain regions 1–3
CB: cerebellum
CX: cortex
DAPI: 4',6-diamidino-2-phenylindole
DEA: diethanolamine
DG: dentate gyrus
EDTA: ethylenediaminetetraacetic acid
EEA1: early endosome antigen 1
flAPP: full length amyloid-β precursor protein
flSez6: full length seizure protein 6
flSez6L: full length seizure 6-like protein
GR: granular layer
HP: hippocampus
HP-β-CD: hydroxypropyl-β-cyclodextrin
LAMP1: lysosomal-associated membrane protein 1
LBPA: lysobisphosphatidic acid
MO: molecular layer
NPC: Niemann-Pick type C disease
NPC1: Niemann-Pick type C gene/protein 1
P0: postnatal day 0
P14: postnatal day 14
PBS: phosphate buffered saline
PBS-T: phosphate buffered saline with Tween 20
sAPPt: total soluble fragment of amyloid-β precursor protein
sAPPβ: soluble N-terminal fragment of amyloid-β precursor protein generated by β-secretase cleavage
SDS-PAGE: sodium dodecyl sulfate polyacrylamide gel electrophoresis
Sez6: seizure protein 6
Sez6L: seizure 6-like protein
sSez6: soluble fragment of seizure protein 6
sSez6L: soluble fragment of seizure 6-like protein
TfR: transferrin receptor
Tris-HCl: tris(hydroxymethyl)aminomethane hydrochloride
wt: wild-type
