# Supplementary figures and images for: BACE1-cleavage of Sez6 and Sez6L is elevated in Niemann-Pick type C disease mouse brains
Source: PLoS One. 2018 Jul 6;13(7):e0200344. doi: 10.1371/journal.pone.0200344 (PMC6034874; doi:10.1371/journal.pone.0200344)

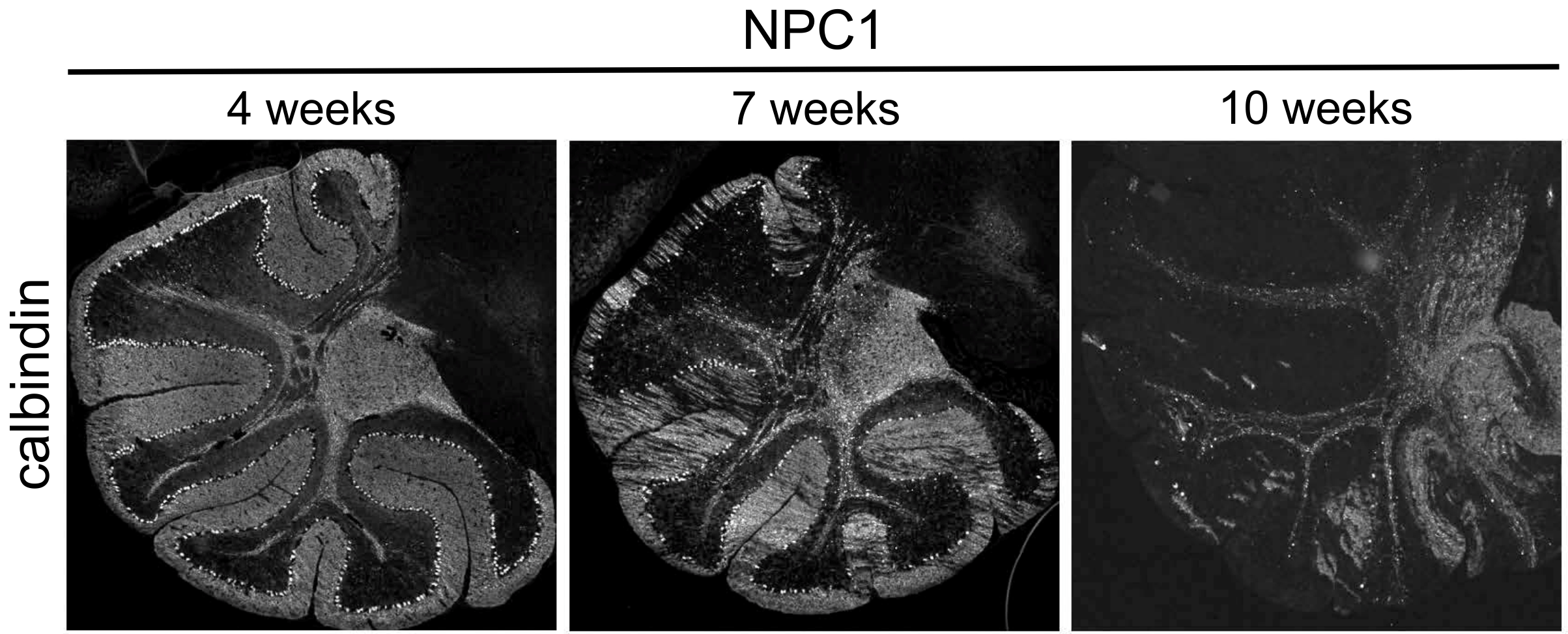

Supplement: S1 Fig — Representative images of calbindin staining in 4-, 7- and 10-weeks old NPC1 mouse cerebellum. The NPC1 cerebella show a partial loss of Purkinje neurons at 7-weeks of age, while at 10-weeks the majority of Purkinje cell immunoreactivity is lost. (TIF) [file pone.0200344.s001.tif]

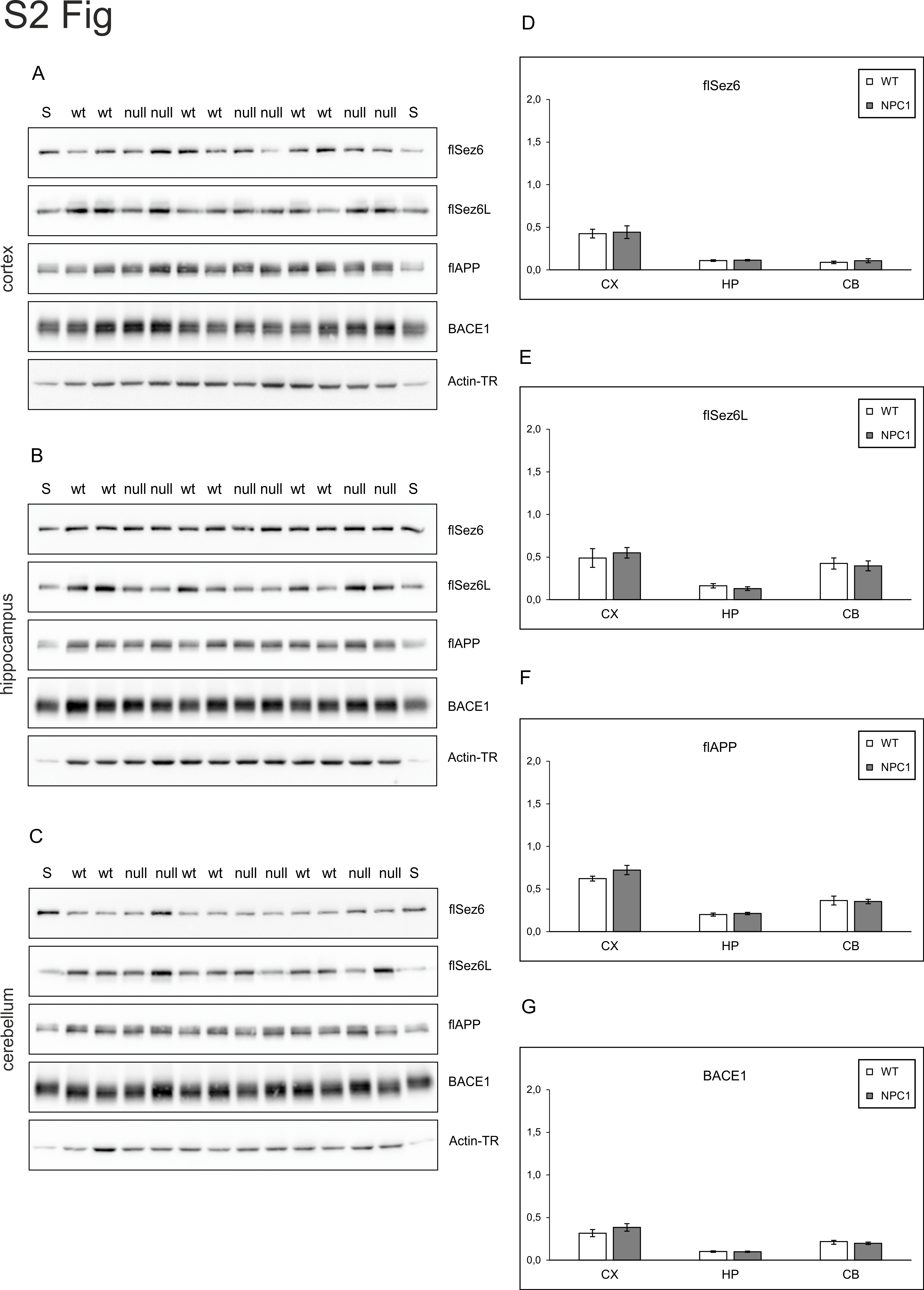

Supplement: S2 Fig — (A-C) Western blot analyses of full-length Sez6 (flSez6), Sez6L (flSez6L), APP (flAPP), BACE1 and actin (Actin-TR) in 1% Triton X-100 (TR) fractions of the cortex, hippocampus and cerebellum collected from 4-weeks old wt (NPC1+/+; N = 6) and NPC1 (NPC1-/-; N = 6) mice. (D-G) Graphs representing quantified protein signals of flSez6 (D), flSez6L (E), flAPP (F) and BACE1 (G) which were normalized against actin (Actin-TR) in the cortex (CX), hippocampus (HP) and cerebellum (CB) of 4-weeks old animals. (TIF) [file pone.0200344.s002.tif]

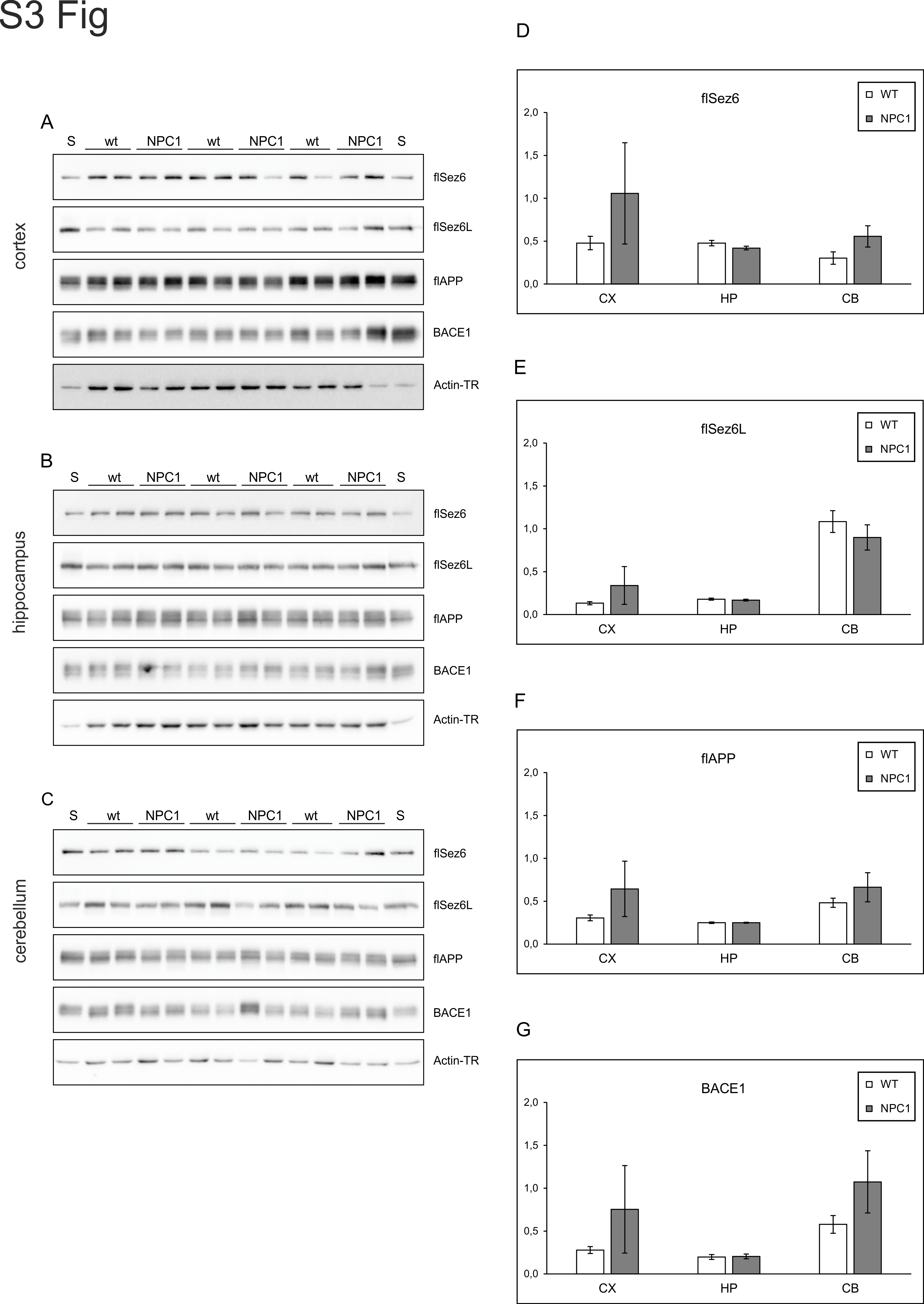

Supplement: S3 Fig — (A-C) Western blot analyses of full-length Sez6 (flSez6), Sez6L (flSez6L), APP (flAPP), BACE1 and actin (Actin-TR) in 1% Triton X-100 (TR) fractions of the cortex, hippocampus and cerebellum collected from 10-weeks old wt (NPC1+/+; N = 6) and NPC1 (NPC1-/-; N = 6) mice. (D-G) Graphs representing quantified protein signals of flSez6 (D), flSez6L (E), flAPP (F) and BACE1 (G) which were normalized against actin (Actin-TR) in the cortex (CX), hippocampus (HP) and cerebellum (CB) of 10-weeks old animals. (TIF) [file pone.0200344.s003.tif]

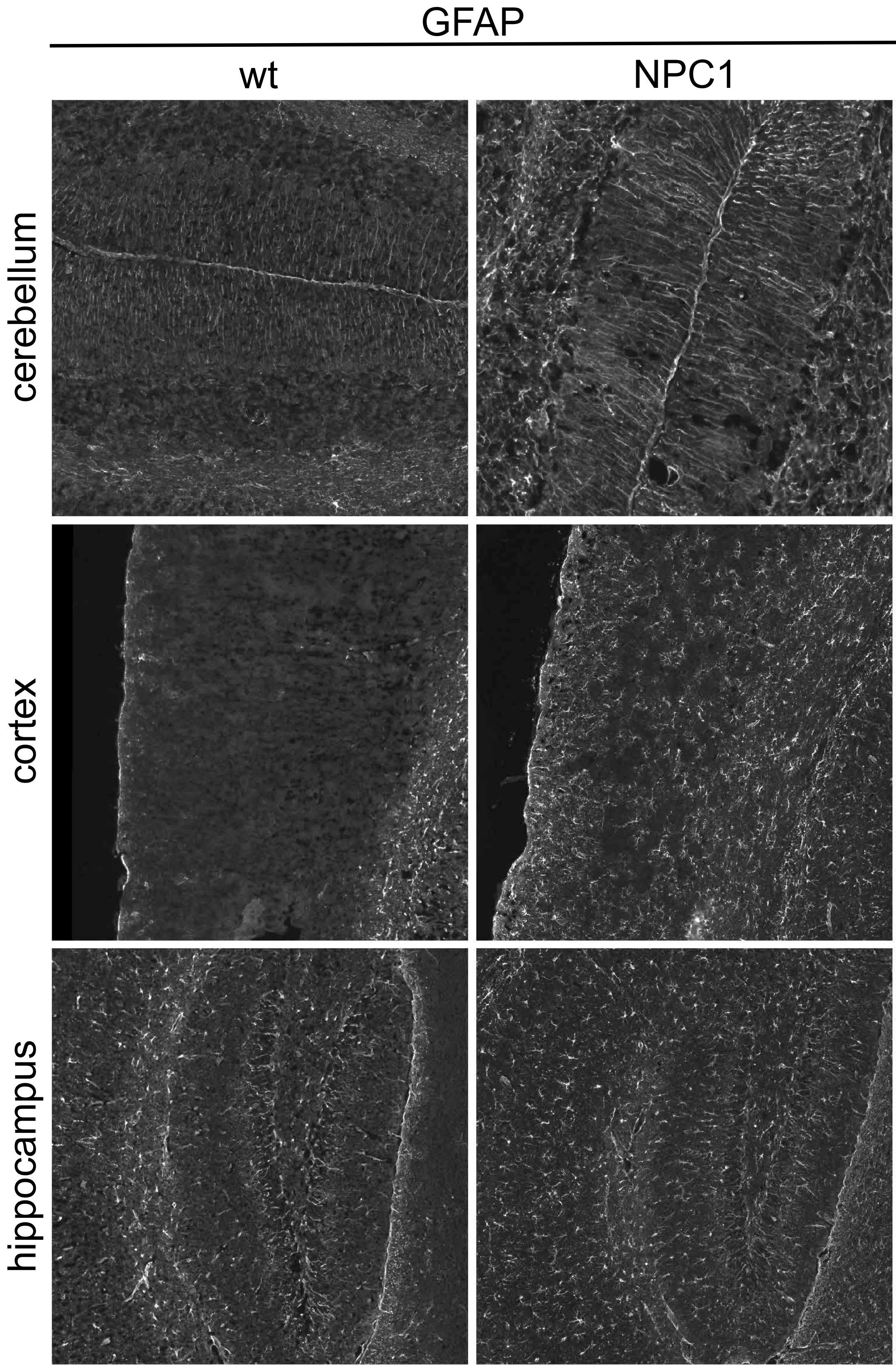

Supplement: S4 Fig — Representative images of glial fibrillary acidic protein (GFAP) staining of cerebellum, cortex and hippocampus. NPC1 mouse brains show a strong immunoreactivity against GFAP indicating profound neuroinflammation, a characteristic feature of NPC disease. (TIF) [file pone.0200344.s004.tif]

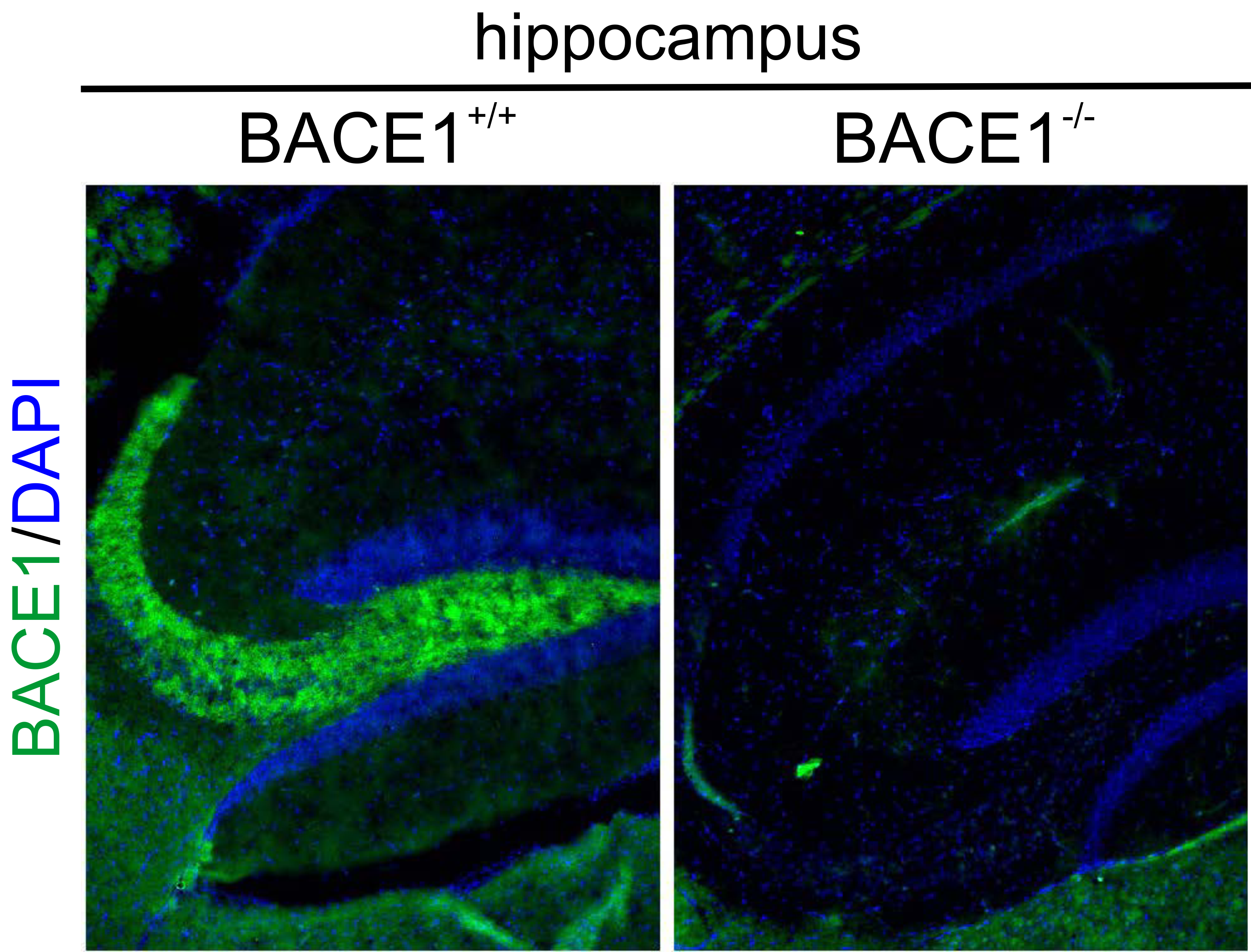

Supplement: S5 Fig — The specificity of the BACE1 antibody (Epitomics, Abcam) was verified in BACE1-/- mouse brain slices. We found BACE1 (green) specific staining only in the mossy fibers in the hippocampus of BACE1+/+ mice. DAPI (blue) was used to counterstain all nuclei. (TIF) [file pone.0200344.s005.tif]

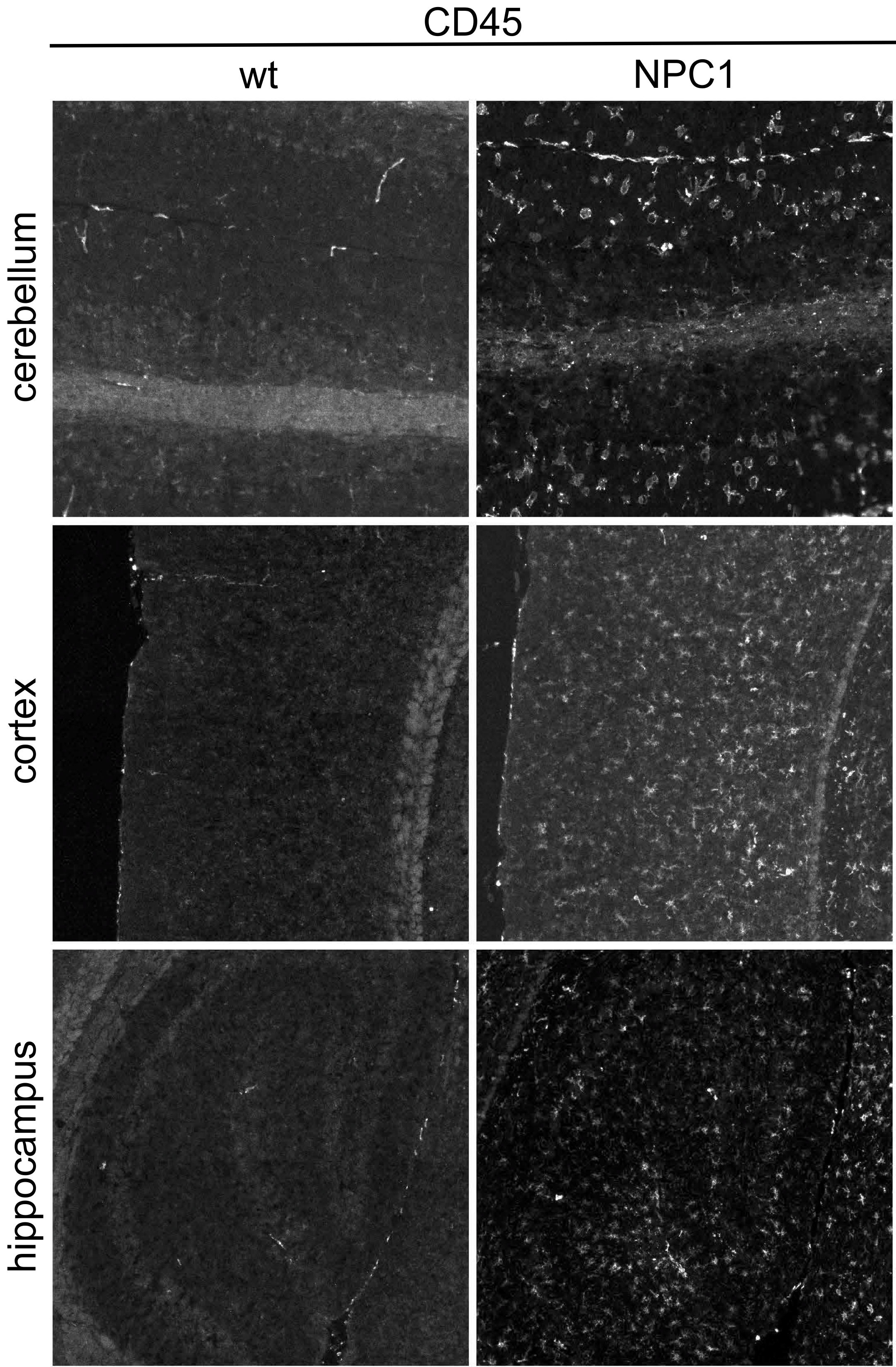

Supplement: S6 Fig — Representative images of CD45 staining of cerebellum, cortex and hippocampus. NPC1 mouse brains show a strong immunoreactivity against CD45 indicating profound neuroinflammation, a characteristic feature of NPC disease. (TIF) [file pone.0200344.s006.tif]
